# *De novo* full-length transcriptome analysis of two ecotypes of *Phragmites australis* (swamp reed and dune reed) provides new insights into the transcriptomic complexity of dune reed and its long-term adaptation to desert environments

**DOI:** 10.1186/s12864-023-09271-y

**Published:** 2023-04-05

**Authors:** Jipeng Cui, Tianhang Qiu, Li Li, Suxia Cui

**Affiliations:** 1 College of Life Sciences, Capital Normal University, Haidian District, Beijing, 100048 China; 2 Beijing Key Laboratory of Plant Gene Resources and Biotechnology for Carbon Reduction and Environmental Improvement, Haidian District, Beijing, 100048 China

**Keywords:** *Phragmites australis*, Combinatorial stress, Full-length transcriptome, Light-harvesting chlorophyll a/b-binding gene, LncRNA, Transcription factor, EST-SSRs, Alternative splicing

## Abstract

**Background:**

The extremely harsh environment of the desert is changing dramatically every moment, and the rapid adaptive stress response in the short term requires enormous energy expenditure to mobilize widespread regulatory networks, which is all the more detrimental to the survival of the desert plants themselves. The dune reed, which has adapted to desert environments with complex and variable ecological factors, is an ideal type of plant for studying the molecular mechanisms by which Gramineae plants respond to combinatorial stress of the desert in their natural state. But so far, the data on the genetic resources of reeds is still scarce, therefore most of their research has focused on ecological and physiological studies.

**Results:**

In this study, we obtained the first *De novo* non-redundant Full-Length Non-Chimeric (FLNC) transcriptome databases for swamp reeds (SR), dune reeds (DR) and the All of *Phragmites australis* (merged of iso-seq data from SR and DR), using PacBio Iso-Seq technology and combining tools such as Iso-Seq3 and Cogent. We then identified and described long non-coding RNAs (LncRNA), transcription factor (TF) and alternative splicing (AS) events in reeds based on a transcriptome database. Meanwhile, we have identified and developed for the first time a large number of candidates expressed sequence tag-SSR (EST-SSRs) markers in reeds based on UniTransModels. In addition, through differential gene expression analysis of wild-type and homogenous cultures, we found a large number of transcription factors that may be associated with desert stress tolerance in the dune reed, and revealed that members of the *Lhc* family have an important role in the long-term adaptation of dune reeds to desert environments.

**Conclusions:**

Our results provide a positive and usable genetic resource for *Phragmites australis* with a widespread adaptability and resistance, and provide a genetic database for subsequent reeds genome annotation and functional genomic studies.

**Supplementary Information:**

The online version contains supplementary material available at 10.1186/s12864-023-09271-y.

## Background

As the global greenhouse effect intensifies, the survival environment of Gramineae plants is becoming more variable and unpredictable under the drastic and frequent intersection of ecological factors such as temperature, radiation, and light intensity. A recent study showed that the desert climate in parts of Central Asia has spread 100 km northward with the global temperature increase in the last 50 years, and the rapid expansion of desertification poses a serious challenge to modern agricultural production systems [1]. The impact on plant growth and development is more severe under compound adversities, especially in ecological environments where natural conditions of high light intensity, high temperature, and drought alternate with other ecological factors [2, 3]. Therefore, in the context of global warming, it is necessary to investigate the survival strategies of plants under natural conditions for long-term adaptation to multi-factor combinations of stress for crop improvement and mitigation of desertification expansion.

*Phragmites australis* is a perennial grass plant belonging to the family Poaceae, which is important for studying plant responses to abiotic stresses because of its complex intraspecific diversity and high phenotypic plasticity, thus *P. australis* has an extensive ecological amplitude and great environmental adaptability [4–6]. In addition, *P. australis* has a wide ecological range and environmental adaptability, with habitats ranging from swamps and lakes to saline deserts, which is important for studying plant responses to abiotic stresses in natural environments. Different ecotypes of reeds are distributed in the oasis-desert transitional zone of northwest China, among which dune reeds (DR), a desert ecotype, is an ideal material for studying the molecular mechanisms of long-term adaptation of Poaceae plants to desert complex adversities [7]. Swamp reed clumps or groups are densely distributed in watercourses with high biomass and dense distribution. In addition, by shielding each other, the leaves of swamp reeds can greatly reduce the strong light and solar radiation stress caused by direct sunlight; Due to the high transpiration and prolonged low humidity environmental characteristics of the desert, and the sparse and uncovered distribution of desert plants, almost all of the leaves of dune reeds have to withstand more intense or direct prolonged high light, solar radiation and drought stress. Therefore, the two ecotypes of reed (DR and SR) have the same climatic environment due to their close distribution distance (about 10 km). However, due to the characteristics of the desert environment, the above-ground part of the two reed ecotypes has to face two habitats with huge differences, and the dune reed has to suffer from more complex and intense multi-factorial combined stress, such as intense and long periods of light, heat, drought and radiation. Due to the large reed genome and complex chromosome ploidy level, such as 2n = 3 × , 4 × , 8 × , 12 × , etc., the assembly of reed genome has been very difficult so far [8]. At present, the lack of genetic resources of reeds greatly limits the research on its stress resistance molecular mechanism.Currently, the shortage of genetic resources for reeds greatly limits research into the molecular mechanisms of their resistance to stress.

The deserts with sparse vegetation and low cloud cover create a harsh habitat with drastic diurnal temperature differences, high sunlight radiation, and extreme drought, and plants exposed to such environments are exposed to a multifactorial cross-section of abiotic stresses dominated by both high temperature and high light stress. It has been shown that when plants are stressed by high light or high temperature or a combination of both, they directly damage chloroplast photosystem II (PSII) and hinder their self-healing process [9–12]. Light energy is not only a direct source of energy for plant material metabolism but also closely related to plant growth and development. However, excess excitation energy in chloroplasts can induce large amounts of reactive oxygen species (ROS) production, and causing damage to the stability of photosynthetic system reaction centers [13, 14]. The light-harvesting chlorophyll a/b binding protein (Lhc) family of proteins has a central role in maintaining the structural stability of vesicle membranes, repairing of the PSII system, and energy transfer, and is involved in photooxidative protection under various stress conditions [15–17]. In higher plants, the Lhca and Lhcb protein subfamilies, located in chloroplast PSI and PSII, respectively, act as pigment-binding proteins of the light capture complex and can regulate the efficiency of light energy capture and transfer in PSI and PSII by controlling the level of excitation light in the photosystem in a balanced manner [18–20]. In addition, when the excitation energy in the photoresponse center exceeds the photosynthetic system load, the plant activates and exacerbates its own defensive energy dissipation photoinhibition phenomenon [21, 22]. Under the cross-stress of multiple ecological factors, how can plants balance the coordination between energy and survival while ensuring the normal function of the photosynthetic system?

With the continuous upgrading and application of sequencing technologies, transcript reconstruction and annotation based on full-length transcript sequencing, followed by mining gene function at the molecular level, have gradually become research hotspots for species with no or difficult-to-assemble reference genomes, e.g., *Cistanche tubulosa* [23], *Panax japonicus* [24], *Cinnamomum porrectum* [25], *Pennisetum giganteum* [26], *Portulaca oleracea* [27], *Nitraria sibirica* Pall [28]. We conducted the first systematic full-length transcriptome analysis of the above-ground tissues of swamp reeds and dune reeds, revealing the alternative splicing pattern of low complexity in long-term adaptation to complex desert adversity. Furthermore, through differentially expressed gene analysis, we identified a number of TFs that may enhance the resistance of dune reeds to harsh desert environments and revealed an important role for the *PaLhc* family in long-term adaptation to complex desert adversities, including high light, extreme temperature differences and drought. Our results provide a valuable and usable genetic resource for functional analysis of the reed genome and studies of resistance genes.

## Results

### Single Molecule Real-Time (SMRT) sequencing on two ecotypes of reeds using PacBio Sequel

To obtain extensive coverage of the full-length transcriptome, we extracted total RNA from aboveground multi-tissue merged samples of wild plants of both ecotypes of *Phragmites australis* and verified that their quality met the requirements for reverse transcription (Supplemental table: Table S1).

**Fig. 1:**
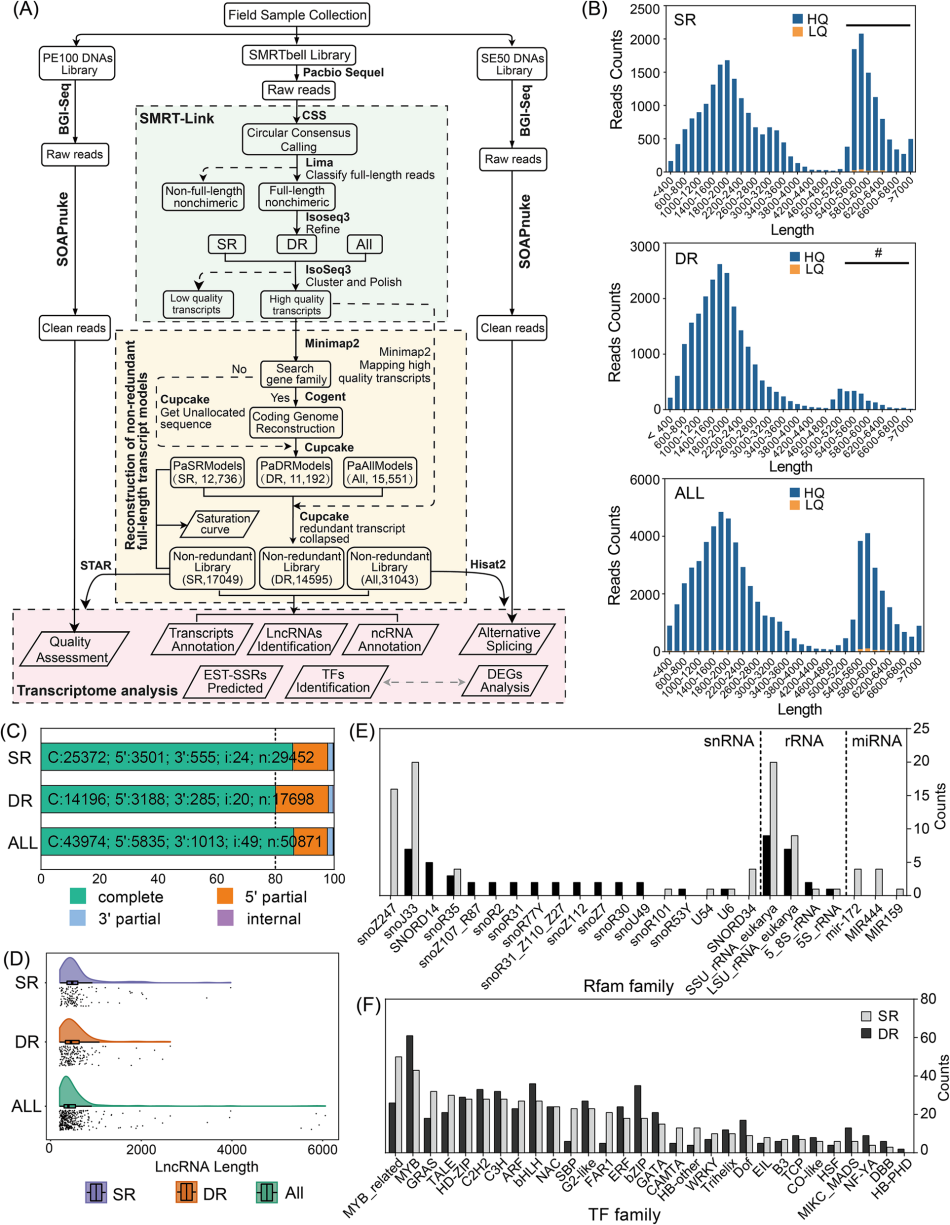
Bioinformatics pipeline and information analysis of non-redundant full-length non-chimeric transcripts in reeds. **A** The pipeline is used for the reconstruction of non-redundant FLNC transcriptome by isoseq 3 without a reference genome. SR: Swamp reed, DR: Dune reed, All: Sample constructed from merged sequencing data from DR and SR. **B** The distribution of reads in three reeds high-quality FLNC transcriptomes after Cluster and polish. HQ: High quality transcripts; LQ: Low quality transcripts."#" indicates the length distribution interval where transcript lengths differ significantly in DR and SR. **C** Summary statistics of predicted ORFs in the non-redundant FLNC transcriptome. The predicted ORF is marked as 'complete' when both start and stop codons exist; as "5' prime partial" when the start codon is missing, and as "3' prime partial" when the stop codon is missing; these ORFs that were missing both were marked as "internal". **D** Length distribution of LncRNAs identified in the non-redundant FLNC transcriptome. In the middle is a box-whisker plot reflecting transcript length dispersion, the upper peaks reflect the density of the length distribution of LncRNAs, and in the lower part is a scatter plot of the length distribution of LncRNAs. **E** Statistics of functional annotation results of ncRNAs in the *P. australis* non-redundant FLNC transcriptome database based on the Rfam-cm database using Infernal software. The bars represent the numbers of transcripts isoforms distributed to different Rfam families. SR and DR shown in grey and black respectively. **F** Distribution of the top 30 TF families predicted in the PlantTFDB. Bars represent the number of transcripts isoforms distributed to different TF families. SR and DR shown in grey and black respectively

SMRT Sequencing was performed of the swamp reed (SR) and the dune reed (DR) using the PacBio Sequel, and we obtained 100,883,402 reads from SR (5 SMRT Cells) and 1,013,317,380 reads from DR (3 SMRT Cells) respectively (Supplemental table: Table S2). To obtain non-redundant full-length non-chimeric (FLNC) transcripts without the *P. australis* reference genome, we developed a bioinformatics pipeline by combining several publicly available software tools (Fig. 1A). A total of 4.44 GB of data volume (1,260,047 reads) was obtained by Iso-seq3, with an average Circular consensus sequencing (CCS) reads of 3,590 bp (Supplemental table: Table S3, Figure S2). When the Refine of CCS reads was performed, the CCS reads data from two ecotypes of *P. australis* were merged to construct a full-length transcripts database (All). Subsequently, we constructed the SR and DR full-length transcripts databases independently after deleting the A01-1 and F02 data from the SR, in order to ensure consistency and comparability in the amount of SR and DR data. After Cluster and Polish, we generated 24,868, 23,936, and 64,021 high-quality (HQ) FLNC transcripts, with N50 of 5,602 bp, 2,261 bp, and 5,488 bp in SR, DR, and All FLNC transcriptome, respectively (Table 1). In addition, we found that the number of transcripts longer than 5,000 bp was significantly lower in DR high-quality transcripts than in SR (Number of transcripts > 5,000 bp: DR, 1,829; SR, 9,364) (Fig. 1B).

**Table 1:** Summary of high-quality FLNC transcriptome after Iso-Seq 3 classification and clustering protocol

Sample	Number of FLNC transcripts	Total base (bp)	N50 (bp)	Mean isoforms length (bp)	Length > 5000 bp	Mean Full-length coverage
SR	24,868	86,688,731	5,602	3,486	9,364	6.90
DR	23,936	49,593,604	2,261	2,072	1,829	6.90
All	64,021	194,853,603	5,488	3,044	19,124	8.04

### Collapsing redundant isoforms and quality assessment

Due to the sensitivity and specificity of the clustering algorithm of Iso-Seq 3 and the susceptibility of the RNA 5' end to degradation during library preparation, some redundant isoforms that are due to technical errors or unrelated to the biological context are still present in the high quality FLNC transcriptome. We reconstructed the non-redundant transcriptome model of *P. australis* through our bioinformatics pipeline, and obtaining three UniTransModels and non-redundant FLNC transcripts databases, respectively (Table 2).

**Table 2:** Statistics of information in the collapsed FLNC transcript database

Sample	Number of non-redundant FLNC transcripts	N50 (bp)	N90 (bp)	Number of Unigenes in UniTransModel	Number of ORFs	Number of complete ORFs
SR	17,030	5,457	1,721	12,728	29,452	25,372 (86.15%)
DR	14,587	2,213	1,206	11,186	17,689	14,196 (80.25%)
All	31,003	5,538	1,657	15,545	50,871	43,974 (86.44%)

The quality of Iso-seq data was assessed by RNA-Seq (BGIseq-500, PE100) data from the same batch of the reed samples. In the All non-redundant transcriptome, 75% of the RNA-seq data had coverage of more than 0.9, and about 7.6% with a coverage of 1 (Figure S3A). As the PacBio Sequel full-length transcriptome is still deficient in detecting low abundance transcripts, ~ 25% of relatively low abundance or rare iso-sequences are not mapped (Supplemental table: Table S4). In the Unigenes—transcripts saturation curve, the saturation curve flattens out as the number of FLNC transcripts increases (Figure S3B). These results indicate that our Iso-seq sequencing volume has largely reached saturation and that the number of transcripts and Unigenes acquired has reached full capacity.

### Open Reading Frame (ORF) prediction, non-coding RNA identification and Transcription factors (TFs) identification in the *P.australis* non-redundant FLNC transcriptome

Using TransDecoder (v5.5.0) to identify potential coding regions in non-redundant FLNC transcripts, and combined with transcript integrity detection, we predicted 29,452, 17,689, and 50,871 ORFs (average amino acid lengths of 398, 397, and 377aa) from the SR, DR, and All FLNC transcript databases, respectively, with the presence of at least 80% of the intact ORFs were identified (Table 2), in addition to 11.5%-18.0% of “5' prime partial”, 1.6%-2.0% of “3' prime partial”, and a very small number of “internal” (Fig. 1C). We subsequently predicted 29,453, 17,689, and 50,871 candidate coding sequences (CDS) in the SR, DR, and All non-redundant FLNC transcriptomes, respectively, and the distribution of predicted candidate CDS lengths was concentrated between 100–800 bp (Figure S4).

In the SR, DR, and All non-redundant FLNC transcriptomes, we identified 126, 169, and 384 long noncoding RNA (LncRNAs), respectively (Supplementary table: Tables S5, S6, S7 and S8). Most of these LncRNAs were distributed around 500 bp in length, but certain LncRNAs with lengths of 2000–6000 bp, and may have the potential coding capacity (Fig. 1D). Interestingly, although more candidate LncRNAs were predicted in DR than SR, most of them were shorter sequences (Average length: SR,547; DR,620). Since the All database was obtained by merged the data of DR and SR after Refine, and the three databases were separately processed using Cluster and Polish, some LncRNAs with lower abundance in SR and DR were retained in the All. Moreover, there are SR:87, DR:54, and All:198 ncRNAs annotated in the known RNA families, respectively (Supplementary table: Tables S5, S6, S7 and S8). Although the number of ncRNAs predicted in the SR was higher, the diversity of predictions in the DR was greater, e.g. snoZ247 was only heavily annotated in the SR, whereas miRNAs were not annotated in the DR (Fig. 1E). We predicted LncRNAs by multiple methods and annotated some ncRNAs in the *P. australis* non-redundant FLNC transcriptome, but further gene mining and experimental validation are needed to determine the function of ncRNAs in *P. australis*.

Transcription factors (TFs) can bind specifically to the promoter sequences of target genes, thereby activating or repressing the expression of downstream target genes at the transcriptional level [29, 30]. We identified 1005, 562, and 557 TFs in the All, SR, and DR FLNC transcriptomes, respectively, and obtained a large number of WRKY (14), bZIP (42), AP2/ERF (33), NAC (42), bHLH (53), C2H2 (60) and MYB superfamily (181) transcription factor family members (Supplementary table: Tables S6, S7 and S8). Among all TF families, the MYB superfamily was the most abundant transcription factor family in the *P. australis*, where the major TFs factors in SR and DR were MYB_related (50, 8.90%) and MYB (61, 10.95%), respectively. In addition, the number of isoforms in the MYB_related, SBP, FAR1, GRAS, and TALE transcription factor families was significantly reduced in the dune reed, whereas it was significantly increased in the MYB, bHLH, and bZIP families (Fig. 1F). Our identification of these transcription factors provides a usable database for studying the regulation of gene expression in *P. australis* under adversity.

### Comprehensive functional annotation of non-redundant FLNC transcripts

**Fig. 2:**
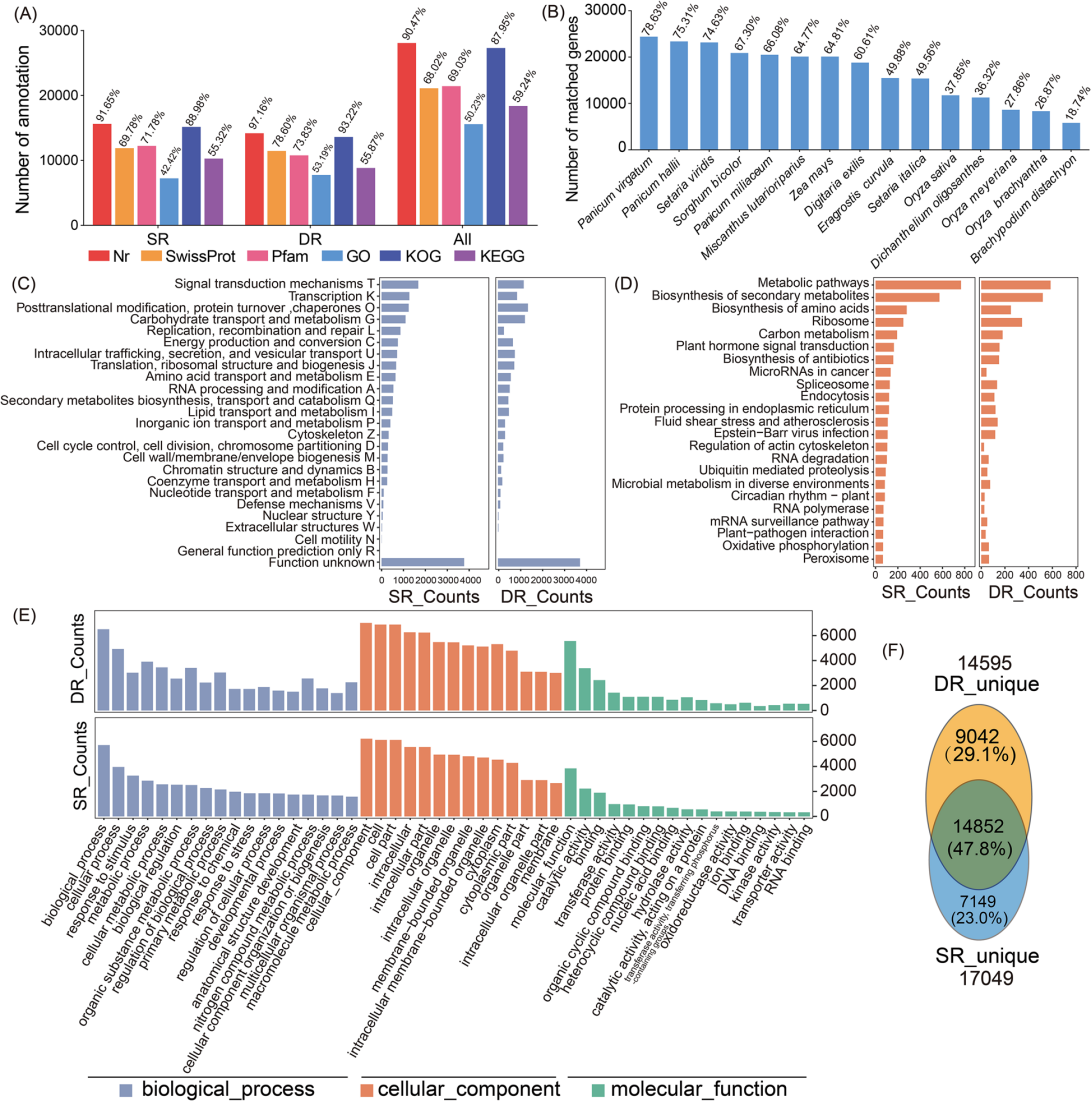
Functional annotation of *P. australis* non-redundant FLNC transcripts. **A** Summary of the functional annotation of All transcriptomes using different databases. **B** The NR homologous species distribution diagram of All FLNC transcripts. **C** The KOG categories of the transcripts. **D** Histogram display of the KEGG pathway categories. The bars represent the number of transcripts distributed in the primary KEGG pathway. **E** Histogram presentation of GO classification. The bars represent the number of transcripts distributed in the different GO functional groups belonging to the three categories. Biological Processes (blue), Cellular Components (red), and Molecular Functions (green). **F** Venn diagram showing the species-unique transcripts in two ecotypes of *P. australis* (swamp reeds and dune reeds*)*. DR_unique is represented as unique transcripts in the dune reed, and SR_unique is represented as unique transcripts in the swamp reed

To obtain comprehensive functional annotations of transcripts for both ecotypes of *P. australis*, we annotated these transcripts by mapping them in the following databases: Nr (NCBI non-redundant protein sequences database), Swiss-Prot (Swiss-Prot Protein Sequence Database), KOG (cluster of orthologous groups of proteins database), Pfam (Collection of protein families database), GO (Gene Ontology) and KEGG (Kyoto Encyclopedia of Genes and Genomes) (Table 3, Fig. 2A, Supplementary table: Tables S6, S7 and S8). Over 90% transcripts of these non-redundant FLNC transcriptomes at least one functional annotation message, with 8427 (All), 4096 (SR) and 4525 (DR) transcripts being annotated in six public databases (Figure S5). BLASTX results based on the Nr database showed that the *Phragmites australis* is closely related to *Panicum virgatum*, *Panicum hallii*, *Setaria viridis*, and *Sorghum bicolor* at the transcripts level (Fig. 2B, Figure S6).

**Table 3:** Statistics on the annotation rate of three FLNC transcript databases

Sample	Nr	SwissProt	Pfam	GO	KOG	KEGG
SR	15,625 (91.65%)	11,897 (69.78%)	12,238 (71.78%)	7,232 (42.42%)	15,170 (88.98%)	9,433 (55.32%)
DR	14,180 (97.16%)	11,471 (78.60%)	10,776 (73.83%)	7,763 (53.19%)	13,606 (93.22%)	8,154 (55.87%)
All	28,084 (90.47%)	21,116 (68.02%)	21,428 (69.03%)	15,593 (50.23%)	27,302 (87.95%)	18,391 (59.24%)

To avoid interference of annotation results by paralogous homologs, we mapped the non-redundant FLNC transcripts to the KOG database. There were 7232 and 7763 transcripts annotated in the KOG database for SR and DR, respectively, with no significant differences in the types of KOG groups involved. In addition, we found large differences in the number of transcript isoforms between SR and DR in certain KOG groups, e.g., dune reeds showed a significant reduction in the number of transcript isoforms in the four KOG groups of Signal transduction mechanisms (T), Transcription (K), Replication, recombination, and repair (L), and Chromatin structure and dynamics (B) (Fig. 2C).

We described differences in the pathway networks of the full-length transcriptomes of the two ecotypes of reeds by mapped non-redundant FLNC transcripts to the KEGG database. For SR and DR, 9,433 and 8,154 transcripts were annotated in KEGG, respectively, of which " Metabolic pathways " and "Biosynthesis of secondary metabolites" were the top two pathways in the *P. australis*. Interestingly, we found that although the number of transcripts annotated to the KEGG database was lower in DR, excluding the "human disease" related pathways, DR has a significantly higher number of transcripts isoforms in certain pathways, such as "Biosynthesis of amino acids (map01230)", "Biosynthesis of antibiotics (map01130)", "Biosynthesis of secondary metabolites (map01110)", and "Carbon metabolism (map01200)" (Fig. 2D).

To distinguish differences in the functional distribution of transcripts in the two ecotypes of *P. australis*, we classified these transcripts by GO analysis. There were 7232 and 7763 transcripts annotated as at least one GO term in SR and DR respectively, and these were grouped into three main functional categories (Biological Processes, Cellular Components, and Molecular Functions). In the category of Biological Processes, "biological process" and "cellular process" were the top two GO Terms. It is noteworthy that the number of transcripts isoforms in DR is substantially increased in the "metabolic process", "cellular metabolic process", "organic substance metabolic process", "primary metabolic process", and "nitrogen compound metabolic process". These secondary metabolism-related transcripts may be beneficial to the long-term resistance of the DR to the complex adversities of the desert. The general trend in the distribution of transcripts in the 'Cellular Component' category was the same for SR and DR, but DR showed a significant increase in the number of transcript isoforms in the 'cytoplasm' category. In the Molecular Function category, transcripts in both reeds were mostly assigned to "molecular function", and "catalytic activity", and more transcripts in DR were assigned to these categories. In addition, to "transferase activity", and " catalytic activity, acting on a protein" and "hydrolase activity" in which the increase in the number of transcripts indicates an increase in hydrolase or catalytic enzyme activity in the DR (Fig. 2E).

We mapped non-redundant FLNC transcripts from DR and SR to All UniTransModel and defined transcripts with more than a 20% difference in reads coverage as species-unique transcripts. The results showed that there were 14,852 transcripts for both DR and SR, with the number of DR-unique transcripts being 9,042 and the number of SR-unique transcripts being 7,149 (Fig. 2F). In the "Biological Process", SR-unique transcripts are mainly assigned to Terms related to plant light response, development and organic or inorganic metabolism such as "long-day photoperiodism", "photoperiodism flowering", and "vegetative to the reproductive phase transition of the meristem"; Whereas DR-unique transcripts have tended to be assigned to multiple protein degradation-related Terms, such as "modification-dependent protein catabolic process", "Ubiquitin-dependent protein catabolic process", and "proteolysis involved in cellular nitrogen compound catabolic process". Among the "molecular functions", SR-specific transcripts were mainly enriched in "molecular transducer activity", "primary active transmembrane transporter activity", "arsenate ion transmembrane transporter activity", while DR was more enriched in "structural constituent of the ribosome", "hydrolase activity". In the Cellular component, the SR-unique transcripts are mainly assigned to the plant vesicle and nuclear chromosome-related Terms, such as "plant-type vacuole", "chromatin", "nuclear chromosome part", and "nuclear chromatin"; In contrast, in DR more transcripts are assigned to the "cytosolic part", "mitochondrial part", "ribosome", "endoplasmic reticulum membrane", and "mitochondrial envelope", which are Terms associated with mitochondria, ribosomes, and cytoplasm. Moreover, we found that more transcripts were assigned to the mitochondrial membrane, endoplasmic reticulum membrane, and extra-nuclear membrane in DR. The species-unique transcript enrichment results revealed high activity of biochemical processes including protein degradation, synthesis, and translocation in DR in response to combinatorial stress in the desert, and that the membrane system may play an important role in this process (Figure S7).

### Expressed sequence tag-SSR (EST-SSR) identification and primer development

**Fig. 3:**
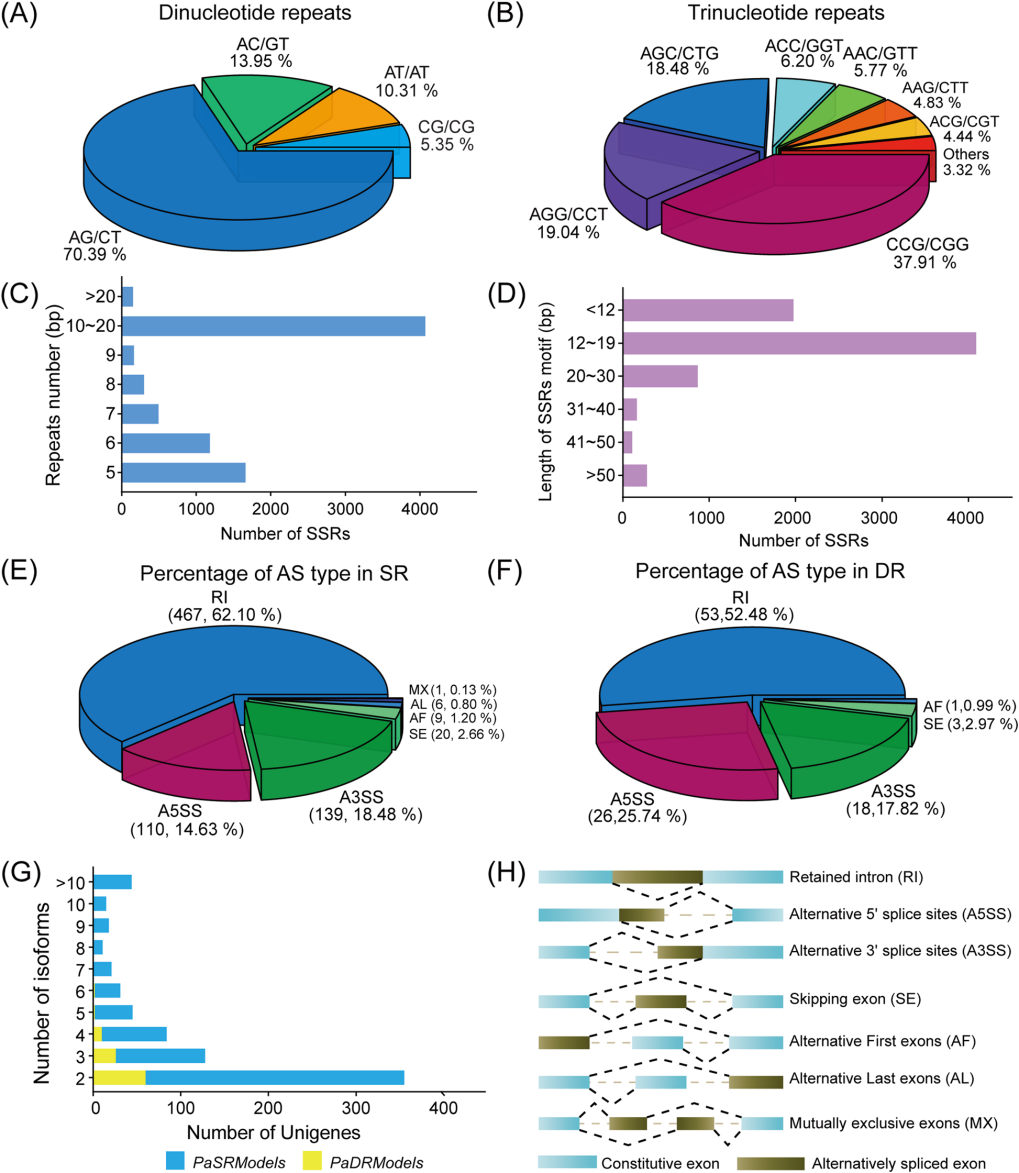
Identification and analysis of expressed sequence tag-SSR (EST-SSR) and alternative splicing (AS) events in the full-length transcriptome. **A**, **B** Distribution of Dinucleotide and Trinucleotide repeat sequence types in All UniTransModels. The different coloured sectors indicate the percentage of different EST-SSR types. **C** Distribution of the number of different repetitive motifs of EST-SSR in the *P. australis*. **D** Statistics of SSRs with different repeat sequence motif lengths. **E**, **F** Pie charts show the percentage of different alternative splicing types in SR and DR, respectively. The different colors of the sectors indicate the percentage of different alternative splice types. **G** Histogram showing Unigenes with different numbers of isoforms in SR vs DR. **H** Alternative splicing pattern diagram. The constitutive exon is represented by a blue block and the alternatively spliced exon is represented by a brown block

SSR (Simple Sequences Repeat) is a class of tandem repeats consisting of a few nucleotides as repeat units up to tens of nucleotides, also known as microsatellite sequences. A total of 8032 potential SSR loci were identified by MISA in the All UniTransModels, distributed among 5530 Unigenes, of which 10.58% (1645) had two or more SSR loci and 3.52% (548) contained the composite SSRs (Supplementary table: Table S9). The frequency of EST-SSR loci in All UniTransModels (ratio of Unigenes containing SSR loci to the total number of Unigenes) was 35.56% and the frequency of occurrence (ratio of the number of SSRs to the total number of Unigenes) was 51.65%, with an average of one EST-SSR locus present every 6076 bp (Table 4). EST-SSR loci were abundant in All UniTransModels, and the number of SSR loci decreased with increasing nucleotide repeats in the SSR motifs. All six types of one to six nucleotide repeats could be detected and their percentages were 46.22%, 21.86%, 28.90%, 1.68%, 0.78% and 0.56%. Single nucleotide, Dinucleotide, and Trinucleotide repeats were the dominant motifs for SSR loci in All UniTransModels, with these EST-SSR types accounting for 96.97% of SSR loci. The Single nucleotide repeats (46.22%) were the major motif type in the All UniTransModels, followed by Trinucleotide repeats (28.90%). And the Dinucleotide repeat motif AG/CT (70.39%) was the predominant EST-SSR type (Fig. 3A), and the trinucleotide repeat motif was mainly CCG/CGG (37.91%) (Fig. 3B, Supplementary table: Table S5). We divided the SSR loci into six groups according to repeat motif length, and the average length of EST-SSR repeat motifs was 18.27 bp. Among them, EST-SSR with repeat motif lengths of 12–19 bp were the most common, accounting for 54.66% (4091) of the overall, and the least number of repeat motifs with the length of 41–50 bp was only 128 (1.41%). In addition, 26.38% (1974) of EST-SSR repeat motifs less than 12 bp were present in reeds (Fig. 3C-D). With 651 low-level repeat motifs (Di- and Tri-nucleotide repeats) ≥ 20 bp in length obtained in the transcriptome of reeds, we speculate that these are abundant and have a high potential for polymorphic studies. EST-SSR motifs containing single nucleotide repeats are thought to be associated with a high risk of error during PCR product synthesis, so they should be ignored in subsequent studies [31]. Based on the 8032 EST-SSR loci obtained, a total of 4354 EST-SSR loci were successfully designed with 5709 primer pairs distributed among 4354 Unigenes (Supplementary table: Table S10).

**Table 4:** Frequencies of different EST-SSR repeat motifs types observed in All UniTransModels

SSR motif	Repeats number							Number of SSRs	Percentage (%)
	**5**	**6**	**7**	**8**	**9**	**10–20**	** > 20**		
**Sin-nucleotide**	0	0	0	0	0	3,653	59	3,712	46.22
**Di-nucleotide**	0	613	295	223	130	401	94	1,756	21.86
**Tri-nucleotide**	1,471	528	192	77	37	16	0	2,321	28.90
**Tet-nucleotide**	99	28	6	1	0	1	0	135	1.68
**Five-nucleotide**	51	11	1	0	0	0	0	63	0.78
**Six-nucleotide**	41	3	0	0	0	1	0	45	0.56
**Total**	1,662	1,183	494	301	167	4,072	153	8,032	100.00
**Percentage (%)**	20.69	14.73	6.15	3.75	2.08	50.70	1.90

### Alternative splicing (AS) identification

Isoform-sequencing can overcome the challenge of losing some reads structural information after transcript splicing in reference genome-free species, and directly obtain full-length mRNA transcripts and their precise structural information without assembly [26, 32, 33]. This study, identified 752 and 101 AS events with variable splice sites in SR and DR UniTransModels, accounting for 6.16% and 0.91% of the total number of Unigenes, respectively (Supplementary table: Table S11). Among them, AS events identified in SR were 7.45 times that of DR. The retained intron (RI) was identified as the major AS event in both reeds, with three AS events, RI, alternative 5'/3' splice sites (A5SSand A3SS), accounting for more than 95% of the detected events, with FL (Including Alternative First (AF) and Last (AL) exons) being relatively few and mutually exclusive exons (MX) being the least common AS event (Fig. 3G, Table 5). And the RI events (50%-60%) are the major AS event in reeds, which is consistent with published data from *Arabidopsis*, *Triticum aestivum L*, and *Panicum virgatum* [34–36]. Compared to SR, RI events decreased by 9.62% (isoforms decreased by 12.97%) and A5SS increased by about 11.12% (isoforms increased by 12.61%) in dune reeds (Fig. 3E-F). Meanwhile, 3062 and 267 transcript isoforms were identified in SR and DR, respectively, among which Unigenes with more than two isoforms accounted for 52.80% and 40.60% of the total number of Unigenes, respectively (Table 5). Notably, the percentage of transcripts isoforms with multiple spliced isoforms was extremely low in DR compared to SR, and the transcripts with 5 or more isoforms in DR are only 4.95% (24.60% in SR) (Fig. 3G).

**Table 5:** Isoforms the number statistics of different alternative splicing types in SR and DR

	**RI**	**SS**		**SE**	**FL**		**MX**	**Total**
		**A5SS**	**A3SS**		**AF**	**AL**		
**SR**	1,865 60.91%	520 16.98%	562 18.35%	83 2.71%	18 0.59%	12 0.39%	2 0.07%	3,062
**DR**	128 47.94%	79 29.59%	47 17.60%	11 4.12%	2 0.75%	0	0	267

Unlike the identification of AS events based on genome identification, these AS events use the UniTransModels as a reference

*SE* Skipping exon, *RI* Retained intron, *SS* Alternative 5' (A5) or 3'(A3) splice sites, *FL* Alternative First (AF) and Last (AL) exons, *MXE* Mutually Exclusive (MX) exons

### Differential expression analysis

**Fig. 4:**
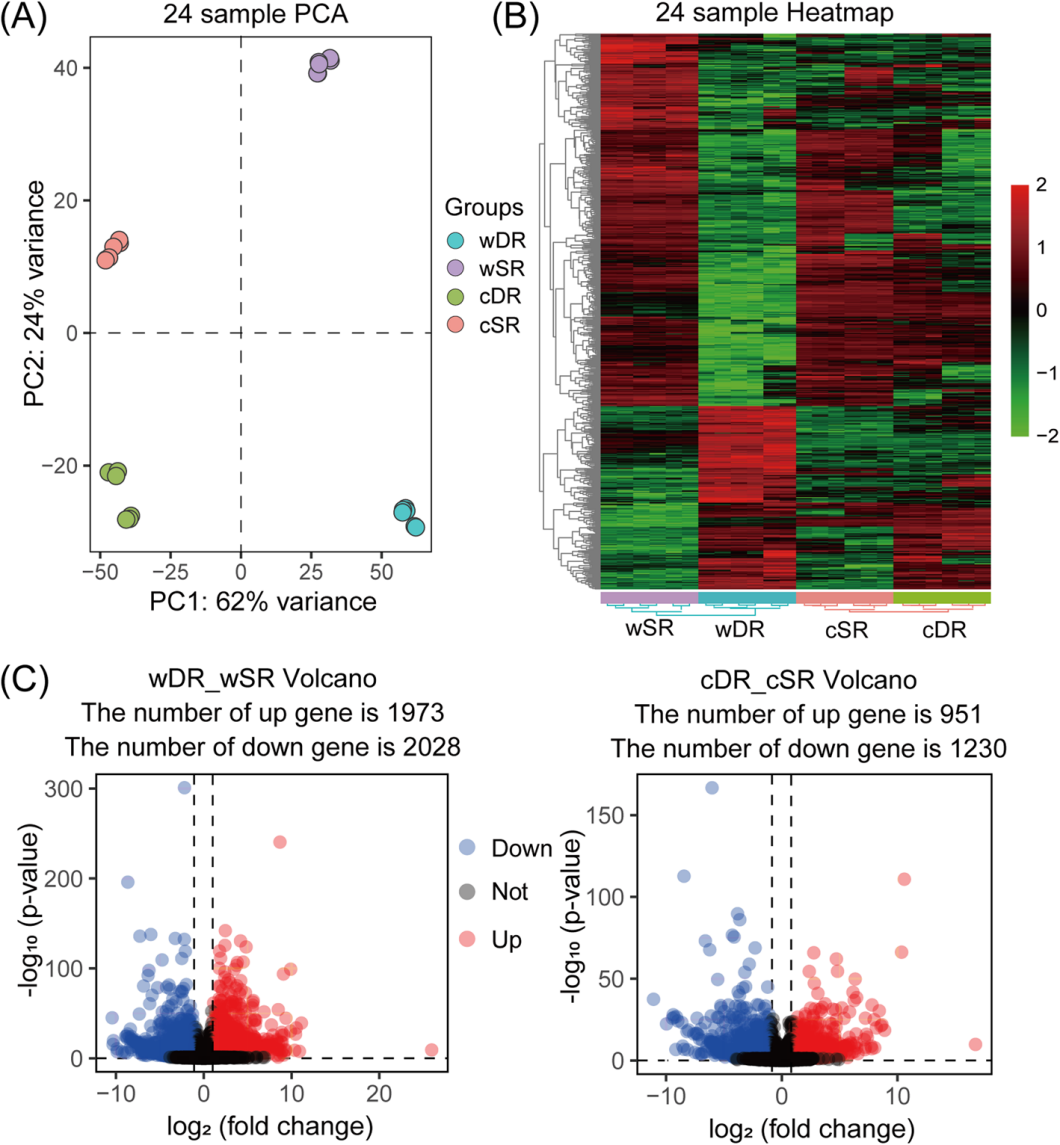
Overview of the RNA-seq data analysis for 24 reed leaf samples. **A** Principal component analysis based on gene expression. Each data point represents an individual reeds of leaf samples. **B** Expressed genes (top 1000) hierarchical clustering diagram in 24 reeds leaf samples. The RPKM matrix of the expressed genes was processed by the scale function with centers and scales. **C** Volcano plot of differentially expressed genes in wild-type reeds versus homogenous cultivation reeds, and the genes with significant differential expression are represented by red dots (up-regulated) and blue dots (down-regulated), *P*-value < 0.05, |log_2_ (fold change) |> 1

To investigate the global differences in gene transcription in dune reeds under long-term resistance to typical desert habitats including high light intensity, long sunlight, and high temperature, we performed RNA-Seq analyses on four reeds sample leaves (24 samples including two technical replicates and three biological replicates) of wild-type wSR and wDR as well as cSR and cDR (after seven years of homogenous cultivation). The results of principal component analysis (PCA) (Fig. 4A) and hierarchical clustering heat map (Fig. 4B) of normalised readscounts for all expressed genes showed significant differences in gene transcript levels between the wild-type and homogenous cultivation. After seven years of homogenous culture, the differences in gene transcript levels between DR and SR decreased, but some genes were still differentially expressed. There were 4001 differentially expressed genes (DEGs) in wDR_wSR (P-value < 0.05, |log2(FC)|≥ 1), of which, there were 1973 up-regulated DEGs and 1809 down-regulated DEGs in wDR; And there were 2181 DEGs in cDR_cSR, with 951 up-regulated DEGs and 1230 down-regulated DEGs in cDR (Fig. 4B). After homogeneous cultivation, we found a 45.49% decrease in the number of DEGs (1820), and the presence of 1259 shared DEGs in wDR_wSR and cDR_cSR (Fig. 4C, Supplementary table: Table S12).

**Fig. 5:**
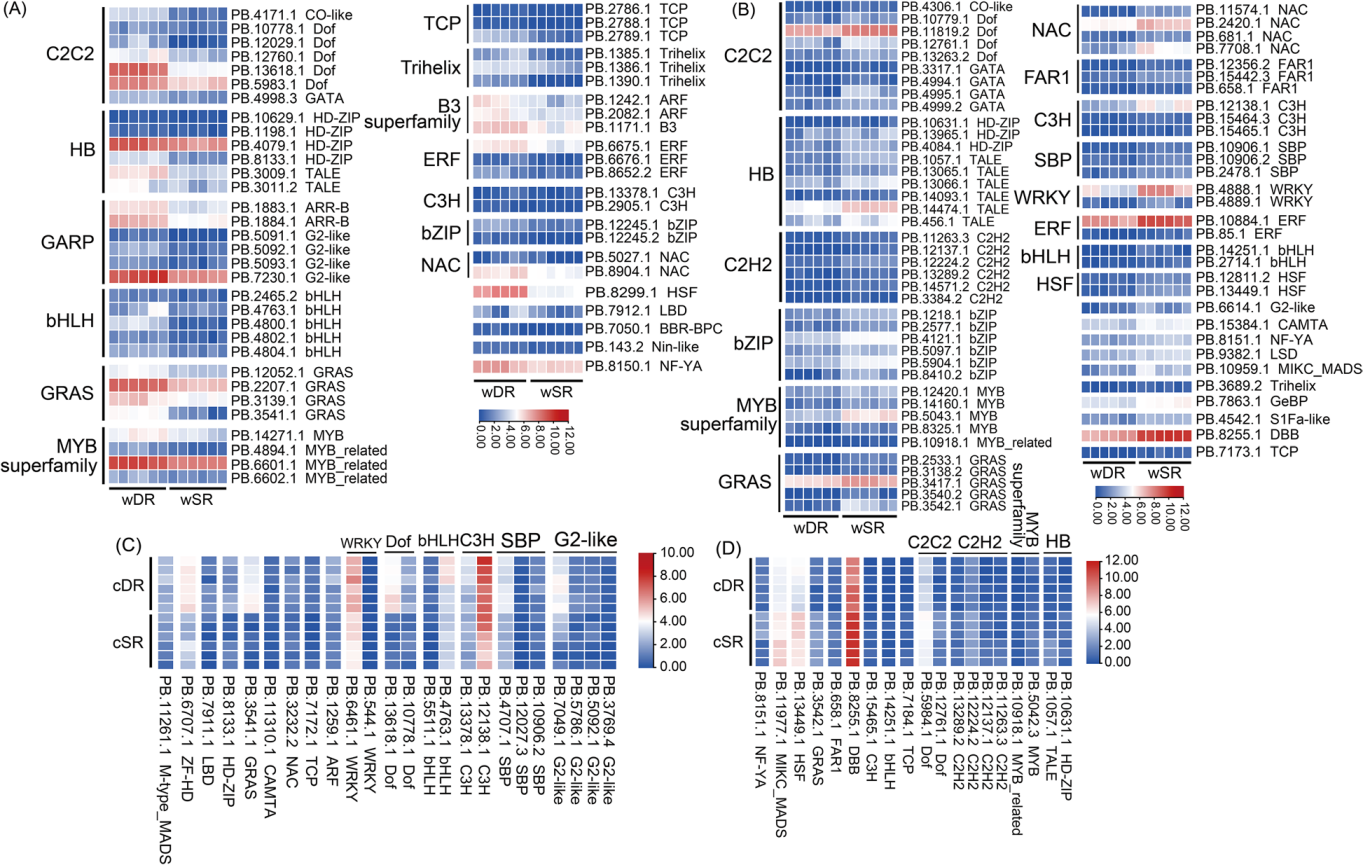
Transcription factor identification among the DEGs. Expression heat maps (**A**) and (**B**) indicate DEGs with up-regulated and down-regulated expression of transcription factors in wDR_wSR, respectively. Expression heat maps (**C**) and (**D**) indicate DEGs with up-regulated and down-regulated expression of TFs in cDR_cSR, respectively. The color scale represents log_2_ transformed counts normalized by RPKM, where blue indicates low expression, the red indicates high expression, and values are expressed as RPKM. *P*-value < 0.05, |log2 (fold change) |> 1

Differential expression analysis of TFs indicated that there were 145 differentially expressed isoforms out of 1005 transcription factors identified in *P. australis* (126 and 42 in wDR_wSR and cDR_cSR, respectively), and the number of differentially expressed transcription factors decreased significantly in *P. australis* of both ecotypes after homogenous culture (Supplementary table: Table S13). Counting the number of differentially expressed members in each TF family, we found a large number of transcript isoforms with up-regulated expression in the bHLH, Dof, G2-like, GRAS, and HD-ZIP families between the two wild-type reeds, and transcript isoforms with down-regulated expression were mostly found in the TALE, bZIP, C2H2, GRAS, and C2C2 family; However, after homogenous culture, a large number of up-regulated members of the G2-like, SBP and Dof families were found, and most of the transcript isoforms were down-regulated in the C2H2 family members (Figure S8). Filtering out these differentially expressed TFs with low expression, we found that in wild-type reeds, Dof (PB.13618.1), HSF (PB.8299.1), GRAS (PB.3541.1) and bHLH (PB.4800.1) expression was significantly up-regulated (log2(FC) 4.19, 3.62, 4.33, 5.32) (Fig. 5A), and significantly down-regulated in DBB (PB.8255.1), WRKY (PB.4888.1), MYB (PB.5043.1), NAC (PB.7708.1) and C3H (PB.12138.1) (log2(FC) -2.66, -2.84, -2.71,—3.22, and -2.73) (Fig. 5B). Moreover, after homogenous culture, we found that the differences in the expression of bHLH (PB.4800.1), HSF (PB.8299.1), WRKY (PB.4888.1), MYB (PB.5043.1), and NAC (PB.7708.1) were no longer significant, but the expression of C3H (PB.12138.1) was adjusted from significantly down-regulated to significantly up-regulated (Fig. 5C-D). The expression adjustment of these transcription factors may play an important role in the resistance of dune reeds to desert complex adversities.

**Fig. 6:**
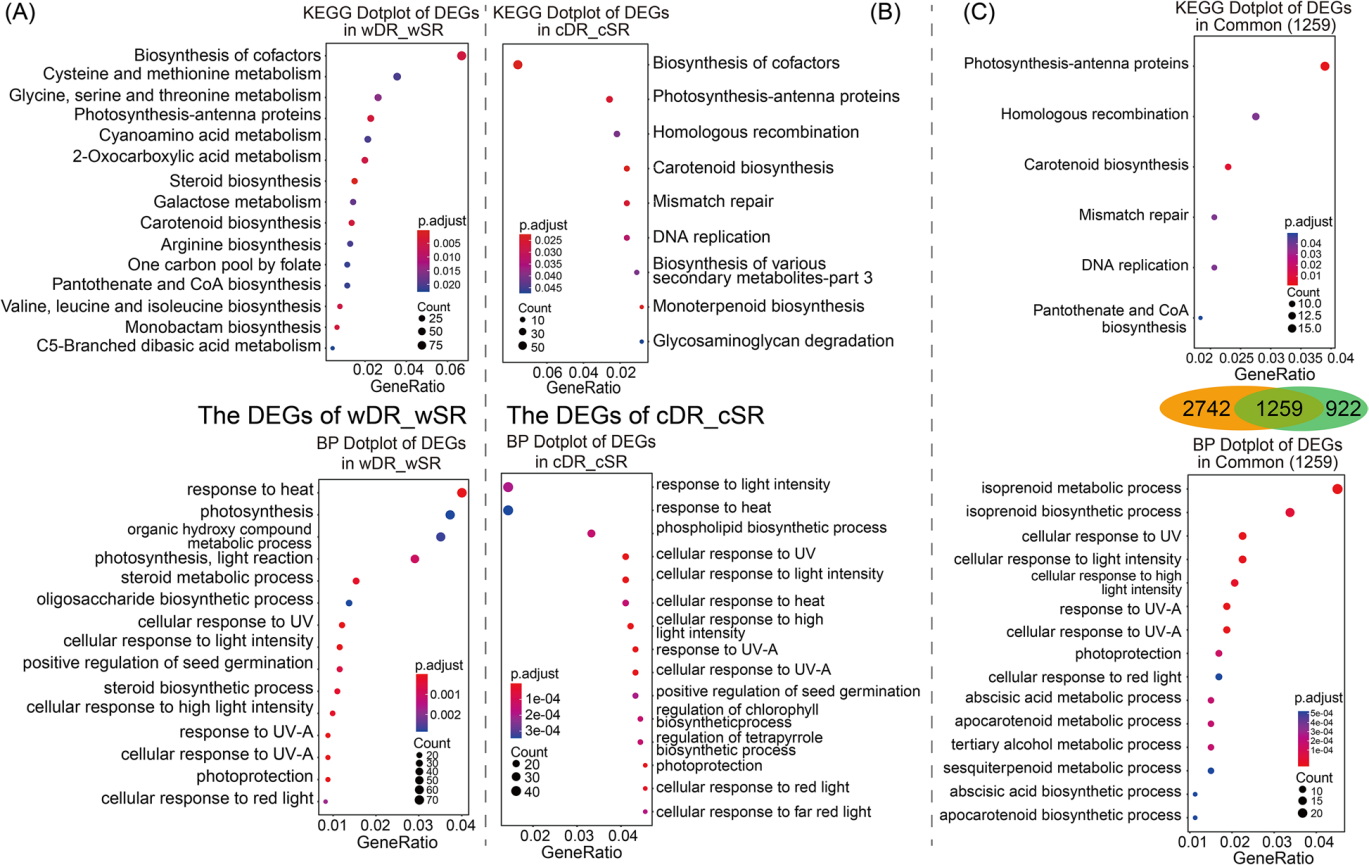
GO (Biological Process) classification and KEGG pathway enrichment analysis of wDR_wSR and cDR_cSR differentially expressed genes. Figures (**A**) and (**B**) show dotplot of GO (Biological Process) classification and KEGG pathway enrichment of differentially expressed genes in two wild-type reeds (wDR and wSR) and homogenous cultured reeds (cDR and cSR), respectively. Figure (**C**) shows a dotplot of GO (Biological Process) classification and KEGG pathway enrichment of commonly Differentially expressed genes (1259) in two wild-type reeds (wDR and wSR) and homogenous cultured reeds (cDR and cSR)

In wDR_wSR, there were certain amino acid metabolic pathways (including 'Cysteine and methionine metabolism', Arginine biosynthesis, 'Valine, leucine and isoleucine biosynthesis', and "Glycine, serine, and threonine metabolism") and "Photosynthesis -antenna proteins" pathway was enriched with a large number of DEGs. In addition, several chloroplast-related biochemical processes were significantly enriched such as "Galactose metabolism" and "Carotenoid biosynthesis". Meanwhile, the results of the Biological Process enrichment showed that most of the DEGs in wDR_wSR were involved in "photosynthesis, light reaction", "photosynthesis", and "cellular response to UV or light intensity" Terms (Fig. 6A-B). Enrichment of common DEGs showed that common DEGs are mainly involved in regulating plant responses to light (UV, light intensity, and carotenoid synthesis), heat, and isoprenoid metabolites, with a large number of common DEGs present in the "photosynthesis-antennae protein" KEGG pathway (Fig. 6C). These results suggest that exposure of the dune reed to the interaction of multiple ecological variables and evolutionary factors in the desert has given rise to important adjustments in its gene expression patterns, that adjustments in pathways associated with multiple light response and protein or amino acid metabolic processes could favour the long-term survival of the dune reed in desert environments, and that these changes may be the result of the ecological domestication of the species.

**Fig. 7:**
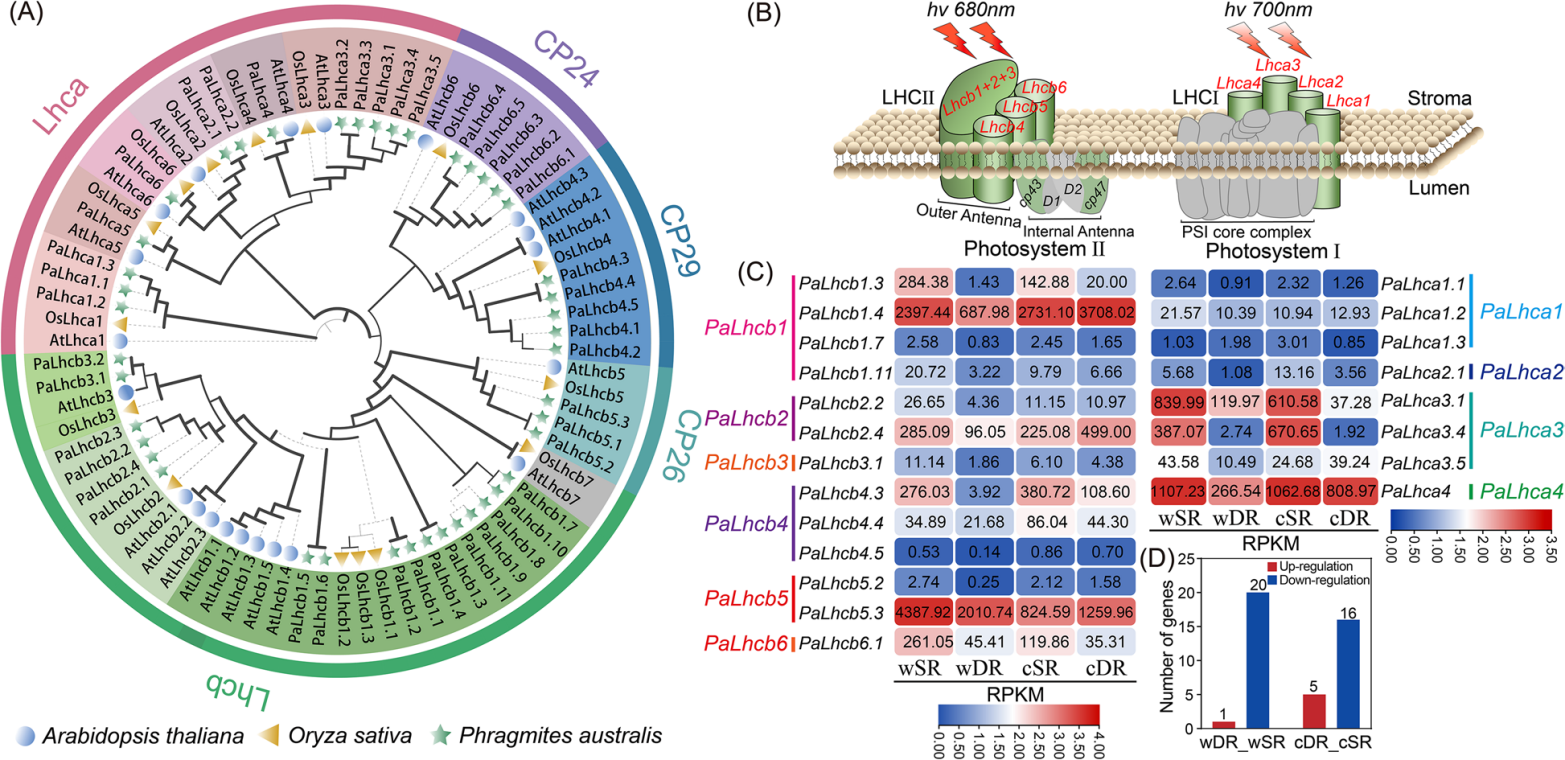
Identification of *Lhc* gene family and differential analysis of *PaLhc* expression in *Phragmites australis*. **A** Phylogenetic analysis of Lhc proteins in *Phragmites australis* (43, Green star), *Arabidopsis thaliana* (21, Blue ball), and *Oryza sativa* (15, Yellow triangle). **B** Distribution of light-harvesting chlorophyll a/b binding protein (LHC) in the photochemical reaction center. In higher plants, PSII light-harvesting antennae proteins include LHCb1-3 as trimers and CP29 (Lhcb4), CP26 (Lhcb5), and CP24 (Lhcb6) as monomers, and PSI light-harvesting antennae proteins are mainly four Lhca proteins (Lhca1-4) that are integrated as monomers in the core complex of PSI [37]. **C** Differential expression of *PaLhc* gene in the *P. australis.* The color scale represents log_10_ transformed counts normalized by RPKM. Where blue indicates low expression, red indicates high expression, and values are expressed as RPKM. P-value < 0.05, |log2 (fold change) |> 1. **D** Statistics on the number of up- and down-regulated members of the LHC family in wild types (wDR and wSR) and homogenous culture (cDR and cSR) reeds. Red bars indicate up-regulation and blue indicates down-regulation

To investigate the role of the "photosynthesis-antennae protein" pathway in the adaptation of reeds to desert environments, we conducted a systematic and comprehensive identification of the chlorophyll a/b binding protein (LHC) gene family based on the *P. australis* candidate protein database. We screened to obtain the final 43 *Lhc* gene family members and named the identified *PaLhc* genes according to their transcript ID number size. The PaLhc family is divided into four main subfamilies: Lhca1, Lhca2-Lhca3, LHCb-CP26, and CP24-CP29, and genes in the same subfamily have similar conserved motifs and necessary conserved domain (Figure S9). The CP24 and CP29 taxa in *P. australis* form a subfamily independent of the major Lhcb protein family. Predictions of the basic physicochemical properties of *P. australis* Lhc proteins indicate that there is some variation in amino acid sequence length and protein properties of the members of the Lhc protein family in the reed, with two main classes of acidic, basic, and hydrophilic different protein compositions (Supplementary table: Table S14). In addition, we found more isoforms in PaLhcb1, and no Lhcb7 subfamily proteins were identified in the All transcriptome (Fig. 7A-B).

RNA_Seq analysis revealed that among the 43 *PaLhc* genes we have identified, 16 family members showed significant differences in expression (|log_2_(FC)|≥ 1, P < 0.05), with *PaLhcb1.4*, *PaLhcb5.3*, *PaLhca3.1*, and *PaLhca4* being highly expressed LHC genes in the *P. australis* (Supplementary table: Table S15). The results showed that multiple LHC family members exhibited down-regulated expression in wild-type and homogenous cultures of dune reeds (Fig. 7D). Compared to wSR, we observed that *PaLhc* expression was down-regulated to different degrees in wDR, except for *PaLhca1.3*, with *PaLhca3.4*, *PaLhcb1.3,* and *PaLhcb4.3* being particularly downregulated (log_2_(FC) -6.86, -7.34 and -5.88, respectively); After homogenous culture, most of *PaLhc* expression still showed a down-regulation trend in cDR, except for *PaLhca1.2*, *PaLhca3.5*, *PaLhcb1.4*, *PaLhcb2.4,* and *PaLhcb5.3*; In addition, we found that the overall expression of *PaLhca4, PaLhcb1, PaLhcb2,* and *PaLhcb4* was dramatically up-regulated after homogenization of DR, while the overall expression of *Lhcb5* was down-regulated (Fig. 7C). These results suggest that *PaLhc* plays an important role in the long-term adaptation of dune reeds to desert adversity.

## Discussion

As an invasive plant with worldwide distribution, the *Phragmites australis* is widely distributed in swampy wetlands and saline desert zones. Deserts are typically a stressful environment with a combination of multiple ecological factors, including alternating combinations of environmental factors such as intense light, drought, high temperature, intense radiation and dramatic temperature differences. The dune reed, which has its habitat in the desert-oasis transition zone, is highly adaptable to desert environments and is a model plant for studying the long-term adaptation of Gramineae to a combination of stresses in the desert in natural conditions [5, 38]. Our previous chloroplast genome study showed that the divergence time of swamp reeds and dune reeds was around 73 Mye [39]. In addition, proteome analysis revealed that net photosynthesis was reduced in dune reeds and that protein content and electron transfer efficiency in photosynthesis-related pathways were significantly downregulated [38, 40, 41].

### Through our bioinformatics pipeline, we obtained non-redundant FLNC transcripts of reeds and performed comprehensive functional annotation and meticulous enrichment analysis

In this study, we obtained three *P. australis* UniTransModel database (All, SR and DR UniTransModels containing 15,551, 12,736, and 11,192 Unigenes, respectively), as well as non-redundant full-length non-chimeric transcript databases (SR, DR, and All containing 17,049, 14,595, and 31,043 transcripts, respectively). Interestingly, we found that the N50 of the DR transcriptome was much lower than that of the SR (SR: 5,602 bp, DR: 2,261 bp), and the number of transcripts with sequence lengths longer than 5000 bp in DR was only 19.53% of that for the SR. These results suggesting that the dune reed tends to transcribe shorter transcripts during long-term adaptation to typical desert environments, which reduces the amount of unnecessary energy loss during post-transcriptional processing (Fig. 1B, Table 2). In addition, the full-length transcripts obtained were functionally annotated through publicly available databases, which provides complete and comprehensive annotation information for the FLNC transcripts of reeds (Table 3, Fig. 2, Supplementary table: Tables S6, S7 and S8). Distribution of homologous species (Nr) showed that *Phragmites* are closely related to *Panicum*, *Setaria*, and *Sorghum* at the transcriptional level (Fig. 2B), which is consistent with results from phylogenetic studies of the chloroplast genome of the grass family [42]. Approximately 40–50% of FLNC transcripts were assigned in the GO & KEGG databases, and the majority of transcripts isoforms in DR were involved in protein synthesis and catabolism, Biosynthesis of secondary metabolites, and catalase activity. We found in the KOG annotation that in DR transcripts in the four major categories of signal transduction mechanisms, transcription, replication, recombination and repair, and chromatin structure and dynamics the transcript’s isoforms were substantially reduced (Fig. 2C). In addition, the functional enrichment of species-specific transcripts showed a substantial increase in DR-specific transcript isoforms associated with mitochondria, cytoplasm, and various cell membrane systems, as well as an increase in transcript isoforms associated with ribosomes and various protein degradation activities.

In summary, the dune reed has reduced the proportion of transcript isoforms in time-consuming and energy-consuming metabolic or regulatory pathways by adjusting its transcriptional patterns, where pathways associated with the cell membrane, protein degradation, and chloroplasts probably facilitate reeds adaptation to desert environments. Our results provide the first systematic description of the full-length transcriptomes of two ecotypes of reeds with significantly different morphology and habitat (SR and DR), and with more comprehensive annotation information, provide a basis for understanding the gene regulatory networks of dune reeds in their long-term resistance to desert abiotic stresses such as drought and strong light in natural environments.

### Based on the full-length transcriptome of reeds, we identified a number of long non-coding RNAs and performed preliminary functional annotation of partial non-coding RNAs in reeds

Non-coding RNAs (ncRNAs) add to the diversity of plant genomes and phenotypes. Still, the synergistic mechanisms among ncRNAs increase the complexity of gene regulatory networks and make it more difficult to study their functional mechanisms. In plants for non-coding RNAs more focus has been placed on LncRNAs, which have been found in several species to regulate plant flowering, abiotic stress response, light signal perception through multiple pathways such as transcriptional repression, chromosome remodeling, selective shearing, and transcriptional enhancers [43–50]. We combined multiple methods to identify and analyze potential ncRNAs in the *P. australis* non-redundant transcript database, obtaining SR: 126, DR: 169, and All:384 LncRNAs, respectively. The increased lncRNAs in DR may help it to resist complex and variable combinatorial stresses in the desert. And based on the Rfam-cm database, some ncRNA functions were annotated (Supplementary table: Table S4). Although these ncRNA functions have not been confirmed, they still provide a positive and useful resource for non-coding RNA research in reeds.

### A large number of transcription factors with potential regulatory roles in the long-term adaptation of dune reeds to desert environments were identified

In plants, MYB [51], bZIP [52], NAC [53, 54], WRKY [55, 56], AP2/ERF [57] transcription factor family members are widely involved in a variety of stress responses, including drought, extreme temperature, and high light. In this study, we analysed the transcription factors contained in the reed transcriptome and identified All: 1005, SR: 562 and DR: 557 candidate transcription factors, respectively. The results indicate that the MYB superfamily is the most abundant family of transcription factors in the *Phragmites australis*. Interestingly, there is a significant decrease in MYB-related and SBP transcript isoforms and a significant increase in MYB, bHLH, and bZIP transcripts in dune reeds. Additionally, we identified 145 differentially expressed transcription factors whose regulation by these TFs could help the dune reed improve its efficiency in regulating stress-related target genes under conditions of energy and material deprivation.

### We identified and systematically characterized for the first time the expressed sequence tag-SSR (EST-SSR) present in Phragmites australis transcripts, and designed EST-SSR primers

Compared to genomic SSR (G-SSR), EST-SSR allows the targeting of markers to genes with actual functions, which is not only more conserved in terms of simple repetitive sequences, but also has good accessibility and versatility, and is therefore important in the study of genetic diversity of gene functions in populations [58–61]. We have revealed for the first time the distribution characteristics of EST-SSRs in the All UniTransModels, identifying a total of 8032 potential EST-SSR loci in 5530 pseudogene transcripts and successfully designing 5709 primer pairs (distributed among 4354 Unigenes). The frequency of EST-SSR occurrence in reeds was as high as 51.65%, which is much higher than species such as *Arachis hypogaea* L (7.71%) [62], *Zelkova schneideriana* Hand.-Mazz (6.04%) [63], *Elymus sibiricus* (6.55%) [64], *Bromus cathartics* [65], Carex L (24.11%) [66]. Among the single nucleotide repeat motifs, the A/T repeats (2,556) were 2.21 times higher than the C/G repeats (1,156), which may be due to the higher number of polyA in the *P. australis* transcript. The mutation of sin-, di-, tetra- or pentanucleotides SSR in the coding region of the gene result in frameshift mutations and affect the structure and function of the protein [67]. We found that sin-, di- and Tri- nucleotide repeats (96.97%) were the dominant motifs for EST-SSR loci in the All UniTransModels, with sin- and di- nucleotide repeat types accounting for 68.08% of the overall number of SSR motifs, which may indirectly account for the complexity and great variation in reeds ecotypes. SSR motif length polymorphism is the driving cause of the differential variation of different types of SSR loci, and its polymorphism is higher when the repeat motif length SSR ≥ 20 bp, in addition, the polymorphism of low-level motif SSR is considered to be generally better than that of high-level motif SSR, so this part of SSR loci was focused on in this study [59]. We have designed the first EST-SSR marker database for All UniTransModels, which will be an important reference for the in-depth assessment of genetic diversity and phylogenetic relationships among reeds species.

### The dune reed reduces transcriptome complexity by massively reducing the occurrence of alternative splicing (AS) events

Alternative splicing (AS) can produce a large number of different mRNA splicing isoforms by alternative splicing of the same pre-mRNA [68]. Previous studies have shown that Alternative splicing in plants plays an irreplaceable role in plant responses to abiotic stresses such as light, drought, and temperature, regulation of the biological clock, and metabolic regulation [26, 69–74]. However, the transcripts produced by alternative splicing often contain premature termination codon (PTC +), which need energy to be degraded through the nonsense-mediated decay (NMD) pathway [34, 69, 75]. In addition, transcripts generated by certain RI events often contain introns characteristic of protein-coding exons (exitrons). Due to the frequent presence of intrinsically disordered proteins (IDPs) or intrinsically disordered regions (IDRs) in the translated products of Exitrons and certain AS isoform, the translated proteins lack a fixed three-dimensional structure or have no any specific function [76, 77]. Although these AS events increase protein substrate diversity and regulatory capacity [69, 74], the translation and degradation of these PTC + containing transcripts, and potentially the degradation of proteins containing IDPs and IDRs, add unnecessary or additional material and energy consumption. Our results showed that the frequency of AS events, the number of isoforms, and the proportion of transcripts with multiple isoforms were substantially reduced in DR compared to SR (Fig. 3).

In addition, transcripts generated by retention intron (RI) events, which are the most abundant in variable splicing in plants, are mostly stored in the nucleus and only function under specific stresses or periods, or degraded by NMD pathways [78, 79]. And dune reeds reduced the proportion of RI (SR: 60.91%, DR: 47.94%) and increased the proportion of alternative 5' splice sites (A5SS) (SR: 16.98%, DR: 29.59%) was increased. When plants are subjected to sudden stresses within a short period, alternative splicing, an important component of the post-transcriptional regulatory machinery, can enable plants to greatly increase the coding potential and protein substrate-specific diversity of certain genes [69, 80–82]. But, to respond to long-term complex stress, plants often need to combine alternative splicing events, and after multi-generational trials and purification selection, they can use variable splicing to efficiently regulate protein diversity or maintain long-term regulatory capacity under limited energy supply [76, 83, 84].

On the one hand, the adaptation of AS events in dune reeds reduces the material and energy costs required for post-transcriptional processing and maximises carbon sequestration efficiency and energy resource allocation; on the other hand, by retaining or adding AS events to specific transcripts, the dune reed optimises transcription patterns and ensures maximum efficiency of transcription under stress, ultimately achieving a cheaper and more efficient way of focusing on way to survive in the extreme desert environment. This may be one of the strategies for long-term adaptation to the multi-factor combination of stresses in the desert. As whole plants were not selected for sequencing, we speculate that additional AS events will be identified when tissue types are expanded and across more developmental stages, but our data still provide data to support alternative splicing studies in reeds under abiotic stress.

### The dune reed reduced light-harvesting chlorophyll a/b binding protein (Lhc) expression to avoid damage to the photosynthetic system from excess excitation energy

The differential expression analysis showed that the number of DEGs decreased by 45.49% between the two ecotypes of reeds after homogenous culture, but a large number of DEGs were still enriched in the “photosynthesis-antennae protein” pathway. In previous proteomic and physiological studies, we found that the dune reed had a lower abundance of multiple proteins involved in the light response and that DR had a lower electron transfer rate and photosynthetic efficiency, but the abundance of rubisco, a key enzyme for CO_2_ assimilation, was not significantly reduced in both ecotypes of reeds [40, 41, 85–88]. In combination with the results of several studies, it appears that plants respond to stresses such as drought, high light, and high temperature by reducing photosynthesis as an important strategy.

In plants, the light-harvesting chlorophyll a/b binding protein (Lhc) is mainly responsible for capturing solar energy and transferring it to the light reaction-center complex in the form of exciting resonance energy. Plants can be transmitted to the light response by regulating the abundance or location of Lhc family members, thereby avoiding the occurrence of photoinhibition and damage under strong light [89, 90]. The identification and analysis of the *Lhc* genes revealed large differences in the number and abundance of its members in different species [17, 19, 20, 91, 92]*.* Using the All non-redundant FLNC transcript database, we identified a total of 43 *PaLhc* family member genes, of which CP29 (*PaLhcb4*) and CP24 (*PaLhcb6*) are relatively evolutionarily independent subfamilies of *PaLhcb1-3*. The results of differential expression analysis showed that wDR downregulated the expression of most members of *PaLhc* compared to wSR, especially the trimer *PaLhcb1-3* (Fig. 5). As excess excitation energy is one of the triggers for ROS and free radicals in the downstream pathway, it has been shown that during the early stages of photomorphogenesis (from darkness to light) in Arabidopsis seedlings, the transcription factors EIN3 and PIF3 can avoid stimulatory damage to plant seedlings by the sudden emergence of intense light by synergistically repressing the transcriptional process of *Lhc* [93]. The dune reed reduces the total amount of excitation light gained into the light reaction centre by reducing *Lhc* expression in long-term adaptation to a multi-ecological combination of stresses in the desert, effectively mitigating the damage to the structure of the photosynthetic system caused by excessive excitation energy due to high light, high temperature and drought, and higher radiation. In addition, there is evidence that plants can influence stomatal sensitivity to ABA and maintain ROS homeostasis in plants under abiotic stress by regulating the expression of certain *Lhc* genes, and that these monomeric proteins often play an important role in photoprotection and non-photochemical quenching (NPQ) [9, 20, 91, 94, 95]. We found that wild-type up-regulated the expression of some members of PaLhcb5 (CP26), PaLhcb6 (CP24) and PaLhca3 in long-term desert-resistant environments compared to dune reeds after homogenous culture, and that these protein monomers may play an important role in resistance to desert complex adversities.

In summary, by reducing the overall expression of light-harvesting chlorophyll a/b binding protein at the expense of total photosynthetic efficiency, dune reeds avoids excessive excitation energy from the upstream of energy input into the photosynthetic system and adapts to the complex and variable desert complex adversity in a cheaper and less energy-consuming way in the long term. On the other hand, during its long-term adaptation to the desert environment, the dune reed up-regulated the expression of some members of the *PaLhc* family related to NPQ or energy escape, which were involved in regulating peroxidation levels and further maintaining the stability of the photosynthetic system. These adaptations in dune reeds play an important role in the resistance to complex desert adversities that include a cross-combination of ecological factors such as light intensity, long daylight, extreme temperature differences, and strong radiation stress.

## Conclusion

In this work, we obtained and deeply analysed the first non-redundant full-length non-chimeric transcriptome of reeds with comprehensive functional annotation. However, because we did not sequentially sample the below-ground parts of the reed, some transcripts expressed in specific tissues (i.e., root and rhizome) or during developmental stages were not obtained, so our results may not fully reflect the adaptability of the reed as a whole. The results show that during long-term adaptation to the desert environment, the dune reed adjusts the frequency and composition of alternative splicing events and reduces transcripts length and transcriptome complexity; On the other hand, by adjusting the expression of *Lhc* family members, the dune reed mitigates photoinhibition and photosystem damage caused by desert stressors such as high light, extreme temperature differences and high radiation in a 'cheap' way. This series of adjustments may be an important strategy for the long-term resistance of dune reeds to the harsh desert environment in natural conditions. Furthermore, the regulation pattern of the dune reed seems to reveal a new research idea for long-term adaptation of plants to survive in extreme environments: by reducing the total value of incoming excitation energy, plants efficiently reduce the stress caused by energy regulation, and plants gain the ability to survive long-term in extreme adversity at the cost of low energy acquisition patterns. Our results provide a valuable genetic resource that is available for studying the molecular mechanisms of stress resistance and the development of molecular markers in reeds.

## Materials and methods

### Plant material collection and RNA isolation

In this study, two wild ecotypes of reeds (wSR and wDR) were collected from two sample sites approximately 10 km apart at the southern edge of the Badain Jaran Desert in Linze County, Gansu Province, China (wSR, 100°7′27″ E, 39°15′39″N; wDR, 100°7′49″ E, 39°21′43″ N), Swamp Reeds (wSR) grow in rivers with year-round water, and Dune Reeds (DR) grow on fixed desert-dunes in the oasis-desert transition (Figure S1). Homogenous cultivation reeds (cSR and cDR) were plants cultivated in the same period and under the same conditions for 7 years in in the College of Life Sciences, Capital Normal University, Beijing, China. On 2 June 2017, Aboveground plant multi-tissue (including the shoot apical meristem, part of the stem, stem nodes, flag leaves, and the first three mature leaves) of 5 wDR and 3 wSR plants were collected for Pacbio Sequel SMRT sequencing. At the same time, the second and third leaves of the four ecotypes of reed (wSR, wDR, cSR, and cDR) were taken for BGIseq500 single-end 50 bp (SE50) or paired-end 100 bp (PE100) sequencing. All samples were quickly packed in tin foil and frozen on dry ice and stored at -80 °C for RNA extraction. Total RNA was extracted using CTAB-pBIOZOL, and DNA contamination in the samples was eluted with 50 μl of RNase-free water. The quality of the extracted RNAs was assessed using an Agilent 2100 Bioanalyzer (Agilent Technologies, Santa Clara, CA, USA).

### cDNA library preparation and PacBio SMRT sequencing

Total RNA samples were sent to Shenzhen BGI Genomics Co., Ltd for cDNA library construction and sequencing. Total RNA is synthesized to first-strand cDNA using Clontech SMARTer PCR cDNA Synthesis Kit. After PCR Optimization, a large-Scale PCR is performed to synthesize second strand cDNA for BluePippin™ size selection. After another large-Scale PCR, the DNA is ready for SMRTbell Template Preparation and sequencing. The libraries were sequenced with a Pacific Biosciences Sequel sequencing instrument.

### Analysis pipeline for PacBio Iso-Seq

High-quality (HQ) full-length isoforms were obtained from PacBio raw data (Subread BAM) using IsoSeq3 software in SMRT Link V7.0.1 (Pacific Bioscience, Menlo Park, CA, USA).

#### Generate CCS (Circular Consensus Calling) from raw subread data

ccs reed.input.subreads.bam reed.ccs.bam –noPolish –minPasses 1

#### Remove primers and barcode

lima reed.ccs.bam primers.fasta reed.fl.bam –isoseq –peek-guess –dump-clips

#### Remove polyA and chimeric sequences, and combine SR and DR libraries to obtain all libraries

isoseq3 refine reed.fl.5p–3p.bam primers.fasta reed. FLNC.bam –require-polya –min-polya-length 20

#### Cluster consensus sequences to generate unpolished transcripts

isoseq3 cluster reed.FLNC.bam reed.unpolished.bam –verbose

#### Polish transcripts using subreads

isoseq3 polish reed.unpolished.bam reed.input.subreads.bam polished.bam –log-file

#### primers.fasta

 > primer_5p.

AAGCAGTGGTATCAACGCAGAGTACATGGGG

 > primer_3p.

AAGCAGTGGTATCAACGCAGAGTAC

The final output of a high-quality (HQ) full-length transcript database is used as sequence files for later analysis.

### Collapsing redundant Isoforms, Identification of alternative splicing (AS)

To remove redundant isoforms in the high-quality (HQ) full-length isoforms dataset, cDNA_cupcake (cDNA_Cupcake v24.3.0, https://github.com/Magdoll/cDNA_Cupcake, accessed on 13 April 2021), and COding GENome reconstruction Tool (Cogent v8.0.0, https://github.com/Magdoll/cDNA_Cupcake, accessed on 13 April 2021) were used in combination to perform further collapsing on the full-length transcript database. First, the high-quality (HQ) full-length isoforms dataset is used to perform preliminary clustering by run_preCluster.py, and generate_batch_cmd_for_Cogent_family_finding.py finely searches for gene families. Next, based on the searched gene family, reconstruct_contig.py was used to reconstruct the gene family gene coding region, and get_seqs_from_list.py was used to obtain the unassigned isoforms. The reconstructed gene coding regions and unmatched isoforms were subsequently merged into a UniTransModels. Finally, the HQ transcripts were mapped to the UniTransModels by Minimap2 [96] software, and then based on the mapping results, the redundant transcripts were further collapsed by collapse_isoforms_by_sam.py to obtain the final FLNC transcripts database. Next, based on the resulting GFF file, alternative splicing (AS) events AS isoforms in the *P. australis* were detected using SUPPA (v2.3) [97] default Settings.

### Next-generation sequencing data analysis

Raw data were cleaned (filtering low-quality reads) using SOAPnuke (v2.0.5) [98] default parameters. To assess the quality of iso-seq data, the pair-end 100 bp (PE100) reads were mapped to three FLNC transcript databases using STAR (v2.7.3) [99]. According to the mapping results, the multi-mapping rates and the data coverage in the All FLNC transcript database were calculated respectively.

For Gene expression quantification and differential expression analysis, single-end 50 bp reads were mapped onto All FLNC transcript databases by Hisat2 [100], and read counts for each isoform were obtained from the mapping results. Thereafter, The R package featureCounts [101] was used to quantify transcript abundance. Isoform abundances are expressed as Reads per kilobase per million mapped reads (RPKM) values. R-package DESeq2 (v1.26) [102] was applied to determine differentially expressed genes (DEGs) from the leaf of reed in different ecotypes with log_2_ |fold change|≥ 1 and P-value < 0.05. R package clusterProfiler [103] was used to enrich the gene function of differentially expressed genes.

To distinguish species-unique transcripts in the two ecotypes of reed, we used Minimap2 [96] to map FLNC transcripts of DR and SR to All UniTransModels respectively, then counted their transcript coverage and identified transcripts with more than 20% difference in coverage as species-unique transcripts.

### Non-redundant FLNC transcriptomes functional annotation

The protein-coding sequences (CDS) in FLNC transcripts were predicted by TransDecoder (TransDecoder v5.5.0, https://github.com/TransDecoder/TransDecoder, accessed on 31 August 2020) software. TransDecoder.LongOrfs was used to detect candidate open reading frames (ORFs) with a minimum length of 100aa in a database of FLNC transcripts. To verify the functional significance of the ORFs in the results, we filtered the obtained ORFs in the Pfam protein domain database using Hmmer (v3.2.1, Available online: http://hmmer.org/, accessed on 19 November 2019) or Swiss-Prot databases by diamond blastp search (E-value ≤ 1e^−5^) [104, 105]. When multiple ORFs were predicted in a transcript, the longest ORF was considered the "representative candidate ORF" and used for subsequent analysis.

Functional annotations of transcripts were aligned using diamond blastx (E-value ≤ 1e^−5^) in Nr (NCBI non-redundant protein sequences) and Swiss-Prot (manually annotated and reviewed protein sequence database) databases [106]. Based on the eggnog V5.0 (evolutionary genealogy of genes: Non-supervised Orthologous Groups) database, eggNOG_mapper v2 is used for GO (Gene Ontology), KEGG (Kyoto Encyclopedia of Genes and Genomes) [107, 108], KOG (cluster of orthologous groups) annotation [109]. Pfam domain annotation information was collected by InterProScan (InterProScan v5.51–85.0, https://github.com/ebi-pf-team/interproscan-docs, accessed on 13 April 2021) to run the scanning algorithms from the InterPro database. R package clusterProfiler was used for gene function enrichment [103].

### Identification of lncRNA and Transcription factors (TFs)

Long non-coding RNAs (LncRNAs) are a group of RNAs with transcripts greater than 200 nucleotides in length that do not encode proteins and are found in a wide variety of organisms. We aligned FLNC transcripts to the Swiss-Prot protein database, filtered any matching transcripts and non-redundant transcripts with CDS equal to or longer than 100 aa, and three lncRNA prediction software CNCI (Coding-Non- Coding-Index), CPC2 (Coding Potential Calculator) and PLEK (Predictor of Long noncoding RNAs and Messenger RNAs based on an Improved k-mer Scheme) were used to predict candidate LncRNAs. To further understand the ncRNA function in reeds, we used Infernal software to functionally annotate ncRNA families from the FLNC transcript database based on the Rfam-cm database.

The plant transcription factor database PlantTFDB v5.0 database (including 165 species and 58 TFs families) is integrated into the PlantRegMap database. Through the PlantRegMap web tools (http://planttfdb.gao-lab.org/), we have Transcription factor identification performed on the remaining full-length transcripts.

### Analysis of simple sequence repeat (SSR) loci and primers design

Potential microsatellites and compound microsatellites were searched and located in All UniTransModels using the microsatellite identification tool (MISA v2.1, https://webblast.ipk-gatersleben.de/misa/, accessed on 25 August 2020), SSR motif minimal repeat identification criteria: single nucleotide base repeats more than 10 times, dinucleotide repeats more than 6 times, three to six nucleosides Acid repeats more than 5 times were defined as microsatellites, in addition, the distance between two microsatellite sequences less than 100 bp was defined as composite microsatellites. The primers of the SSR motif were designed using Primer 3.0 (Primer 3.0 v2.6.1, http://primer3.sourceforge.net), and the main parameters of primer design are as follows:

The length of the flanking sequence of the SSR site is ± 150 bp, excluding some sequences with shorter SSR flanking sequences; the GC base content is between 45 and 55%, and 55% is the optimal parameter; the primer length is between 18 and 25 bp. between, with 23 bp as the optimum length; The annealing temperature (Tm) is between 55 °C and 65 °C, with 60 °C as the optimum temperature, and the difference between the forward and reverse primer annealing temperatures is within 5 °C; The PCR amplification product size is between 100–300 bp; There is no primer-dimer and secondary structure between primers and 1–5 pairs of primers are finally selected for each SSR loci, and the first pair is the optimal primer.

### Identification of Light-Harvesting Chloro a / b-bind gene (*Lhc*) family members in *Phragmites australis*

First, we searched and downloaded 21 AtLhc family protein sequences in Arabidopsis from the TAIR database(https://www.arabidopsis.org/, Araport11), then, AtLhc was used as the query sequence, and 15 OsLhc protein sequences were obtained by blastp in the TIGR rice genome database (http://rice.tigr.org/tdb/e2k1/osal/index.shtml). Next, to identify the members of the Lhc protein family in the *P. australis* candidate protein database, we performed a Blastp (E-value ≤ 1e-5) search with 21 AtLhc protein sequences as query sequences and a hidden Markov model (CB, Chloroa_b -bind, PF00504) used Hmmer (v3.2.1) software (E-value ≤ 1e-5) to search for the *P. australis* protein sequence containing this domain and obtained the *P. australis* candidate LHC protein sequence by taking the intersection of the results of the two retrieval methods. In addition, the reliability and integrity of the chlorophyll a/b binding domain in the *P. australis* Lhc protein sequence were verified in the Pfam database (http://pfam.xfam.org), CDD database (https://www.ncbi.nlm.nih.gov/Structure/cdd/wrpsb.cgi) and SMART database (http://smart.embl-heidelberg.de). Finally, ProtParam (http://web.expasy.org/protparam/) was used to calculate various physical and chemical parameters of the *P. australis* Lhc protein, including molecular weight (MW), theoretical value, and overall average hydrophilicity; Plant-PLoc (http://www.csbio.sjtu.edu.cn/bioinf/plant-multi) was used for protein subcellular localization; DeepTMHMM has been used to predict protein transmembrane helical regions and their topologies [110, 111].

### Sequence alignment, phylogenetic and conserved motif analysis

Lhc protein sequence alignment was performed by mafft (v7.407) and iq-tree (v2.0.3) software was used to construct the phylogenetic tree of the bootstrap of 1000 replicates with the maximum likelihood method. Subsequent visualization and embellishment were performed by iTOL (https://itol.embl.de/). Conserved motifs in Lhc proteins were predicted in MEME (http://meme-suite.org/tools/meme) with the following parameters: The program was set to search for 20 motifs; any number of repetitions; the maximum number of motifs, 15; minimum sites, 2; the optimum width of each motif, between 6 and 100 residues. Finally, TBtools software was used to visualize the structures [112].

## Supplementary Information

**Additional file 1: Supplementary Table S1.** Iso-Seq library RNA quality detection. **Supplementary Table S2.** Summary of Subreads reads in SMRT Cells. **Supplementary Table S3.** Summary of the quality of each library Circular consensus sequencing (CCS) reads. **Supplementary Table S4.** RNA-seq and ISO-seq transcriptome database comparison results. **Supplementary Table S5.** LncRNA prediction and ncRNA annotation statistical results. **Supplementary Table S6.** These public databases showed the annotation results of the SR full-length transcripts. **Supplementary Table S7.** These public databases showed the annotation results of the DR full-length transcripts. **Supplementary Table S8.** These public databases showed the annotation results of the All full-length transcripts. **Supplementary Table S9.** Expressed sequence tag-SSR (EST-SSR) identification in All_UniTransModels using MISA. **Supplementary Table S10.** Primers designed by Primer 3.0 based on EST-SSR motifs in All_UniTransModels. **Supplementary Table 11.** Distribution of alternative splice types in SR and DR.Green fill indicates swamp reed, yellow fill indicates dune reed. **Supplementary Table 12.** Differential expression of genes (DEGs) involved in SR and DR. Blue fill indicates DEGs between the two wild type reeds (wDR and wSR) and grey fill indicates DEGs between reeds (cDR and cSR) after homogenous culture. **Supplementary Table 13.** Differentially expressed genes involved in the identified transcription factors (TFs). Blue fill indicates transcription factors differentially expressed between the two wild type reeds (wDR and wSR) and grey fill indicates transcription factors differentially expressed between reeds (cDR and cSR) after homogenous culture. **Supplementary Table 14.** PaLhc gene family identified in Phragmites australis. **Supplementary Table 15.** Differentially expressed genes involved in the light-harvesting chlorophyll a/b-binding genes.

**Additional file 2: Supplementary figure S1.** Habitats of two wild-type ecotypes of reed in our study. **Supplementary figure S2. **Length distribution of CCS reads in the Pacbio Iso_seq library. **Supplementary figure S3. **Quality assessment of non-redundant FLNC transcript databases. **Supplementary figure S4. **Length distribution of predicted candidate CDS sequences in the full-length transcriptome.**Supplementary figure S5. **Upset plots of annotation results for three full-length non-redundant transcript databases. **Supplementary figure S6. **Distribution of homologous species in the SR, DR and All non-redundant transcriptomes annotated in the NCBI non-redundant protein sequences database. **Supplementary figure S7. **GO function clustering results for DR specific-unique transcripts. **Supplementary figure S8. **Differentially expressed genes in transcription factors identified in *Phragmites australis*. **Supplementary figure S9. **Structural analysis of Lhc family proteins in *Phragmites australis* (43), *Arabidopsis thaliana* (21), and *Oryza sativa* (15).

## Data Availability

The datasets supporting the conclusions of this article are included within the article and as additional files. The raw sequencing reads are available in the NCBI Sequence Read Archive (SRA) BioProject database with the accession number of PRJNA887694 (https://www.ncbi.nlm.nih.gov/sra/?term=PRJNA887694). Other data sets generated in this study will be available from the corresponding author upon request.

## Abbreviations

SMRT: Single Molecule Real-Time
Iso-Seq: Isoform Sequencing
CCS: Circular Consensus Sequencing
Cogent: Coding GENome reconstruction Tool
FLNC: Full-length non-chimeric
ORFs: Open reading frames
CNCI: Coding-Non-Coding-Index
PLEK: Predictor of Long non-coding RNAs and Messenger
CPC: Coding Potential Calculator
EST-SSRs: Expressed sequence tag—simple sequence repeats
AS: Alternative splicing
LncRNA: Long non-coding RNAs
TF: Transcription factor
Swiss-Prot: Swiss-Prot Protein Sequence Database
COG: Clusters of orthologous groups for eukaryotic complete genomes
GO: Gene Ontology
KEGG: Kyoto Encyclopedia of Genes and Genomes
Nr: NCBI non-redundant protein sequences database
Pfam: Collection of protein families database
DEG: Differentially expressed genes
RPKM: Reads per kilobase per million mapped reads
Lhc: Light-harvesting chlorophyll a/b binding protein
