# Supplementary material for: De novo full-length transcriptome analysis of two ecotypes of Phragmites australis (swamp reed and dune reed) provides new insights into the transcriptomic complexity of dune reed and its long-term adaptation to desert environments
Source: BMC Genomics. 2023 Apr 5;24:180. doi: 10.1186/s12864-023-09271-y (PMC10077656; doi:10.1186/s12864-023-09271-y)
Supplement: Supplementary file 2 — Additional file 2: Supplementary figure S1. Habitats of two wild-type ecotypes of reed in our study. Supplementary figure S2. Length distribution of CCS reads in the Pacbio Iso_seq library. Supplementary figure S3. Quality assessment of non-redundant FLNC transcript databases. Supplementary figure S4. Length distribution of predicted candidate CDS sequences in the full-length transcriptome.Supplementary figure S5. Upset plots of annotation results for three full-length non-redundant transcript databases. Supplementary figure S6. Distribution of homologous species in the SR, DR and All non-redundant transcriptomes annotated in the NCBI non-redundant protein sequences database. Supplementary figure S7. GO function clustering results for DR specific-unique transcripts. Supplementary figure S8. Differentially expressed genes in transcription factors identified in Phragmites australis. Supplementary figure S9. Structural analysis of Lhc family proteins in Phragmites australis (43), Arabidopsis thaliana (21), and Oryza sativa (15). [file 12864_2023_9271_MOESM2_ESM.docx]

**
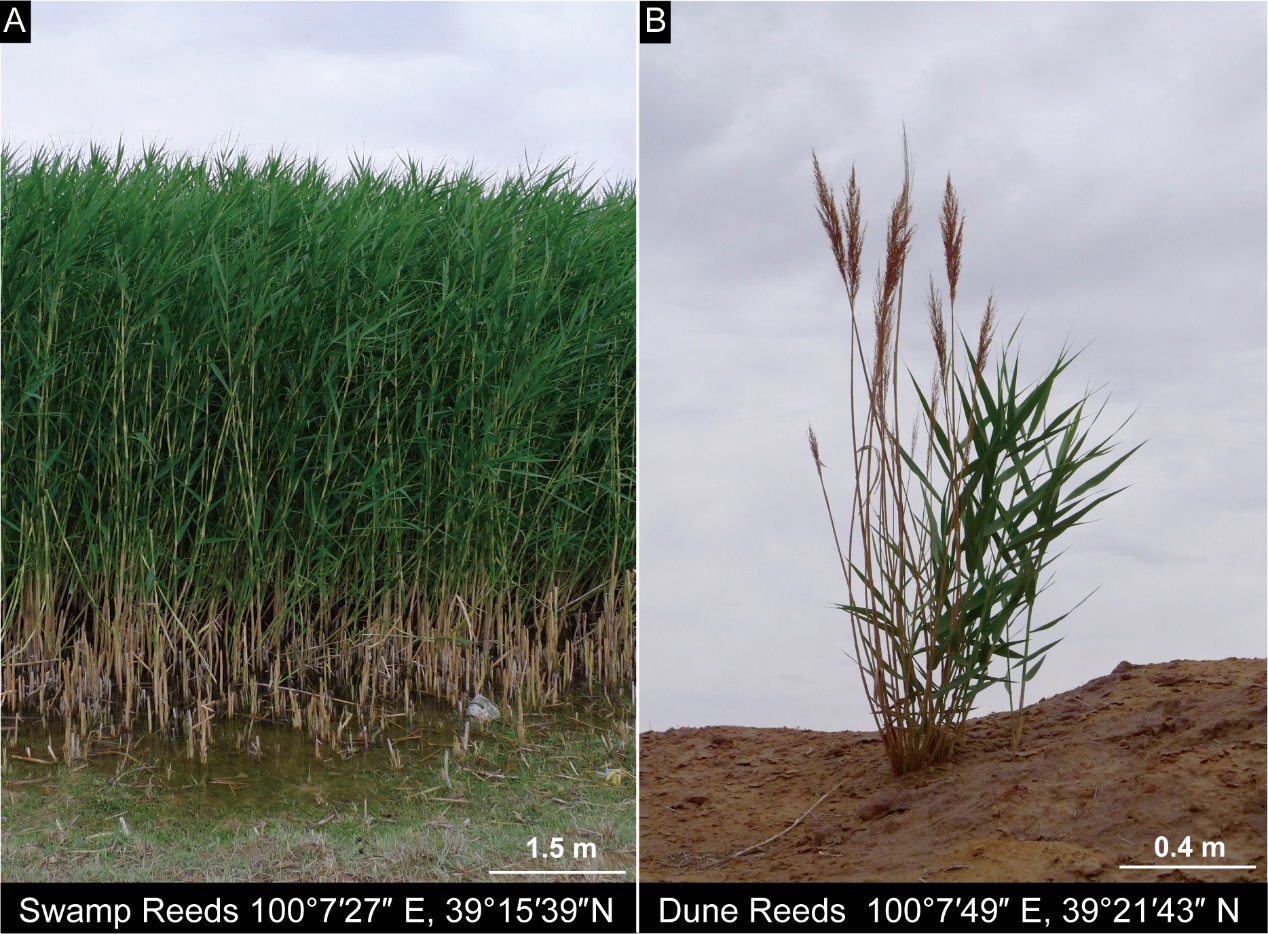
**

**Supplementary figure S1**: Habitats of two wild-type ecotypes of reed in our study. (A) Swamp reed (SR), which grows naturally in river gullies with year-round water; and (B) dune reed (DR), which grows in fixed dunes in the oasis-desert transition zone. The photograph was taken in the area bordering Linze County on the edge of the Badangilin Desert in north-western China.


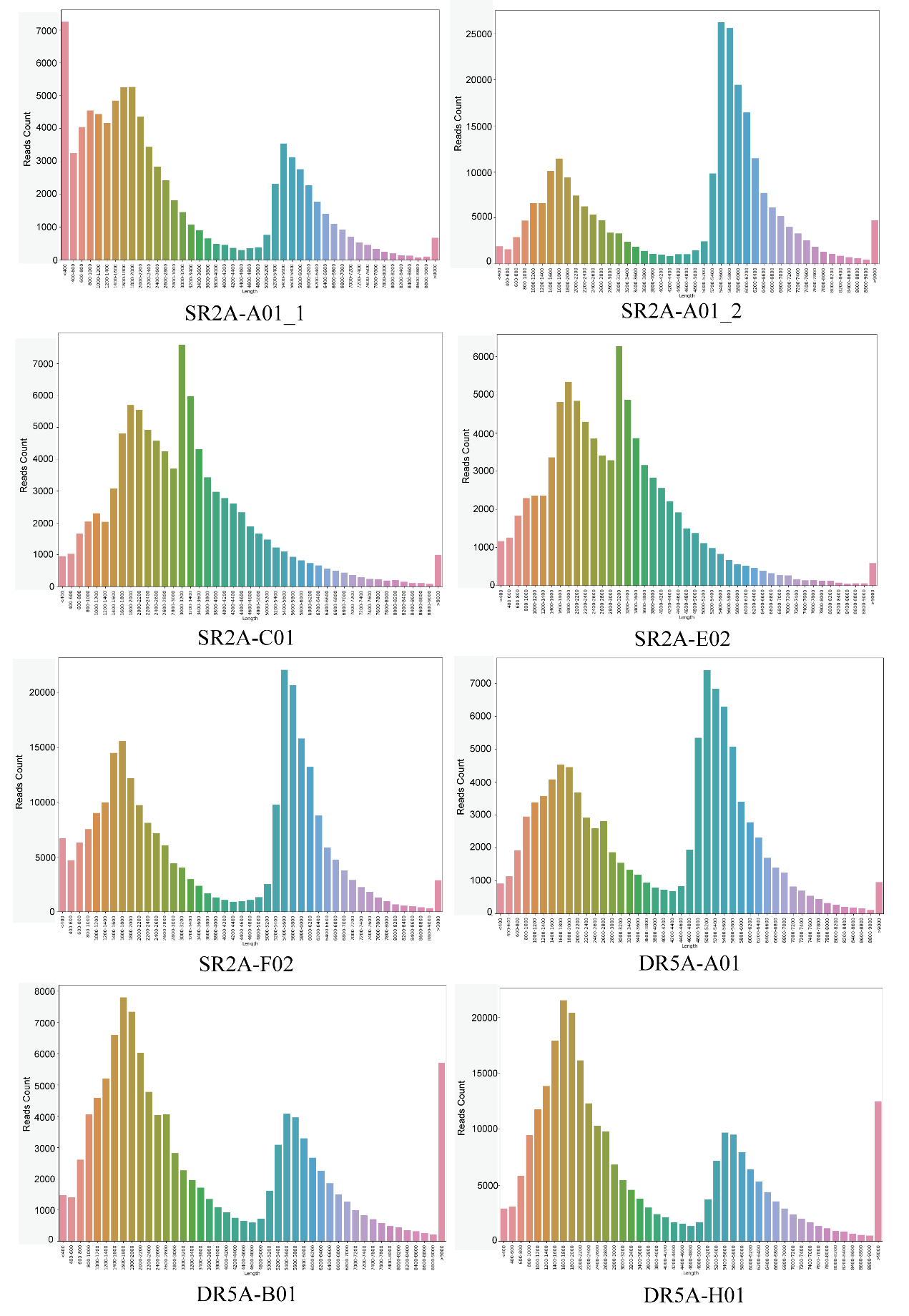


**Supplementary figure S2**: Length distribution of CCS reads in the Pacbio Iso_seq library.

**
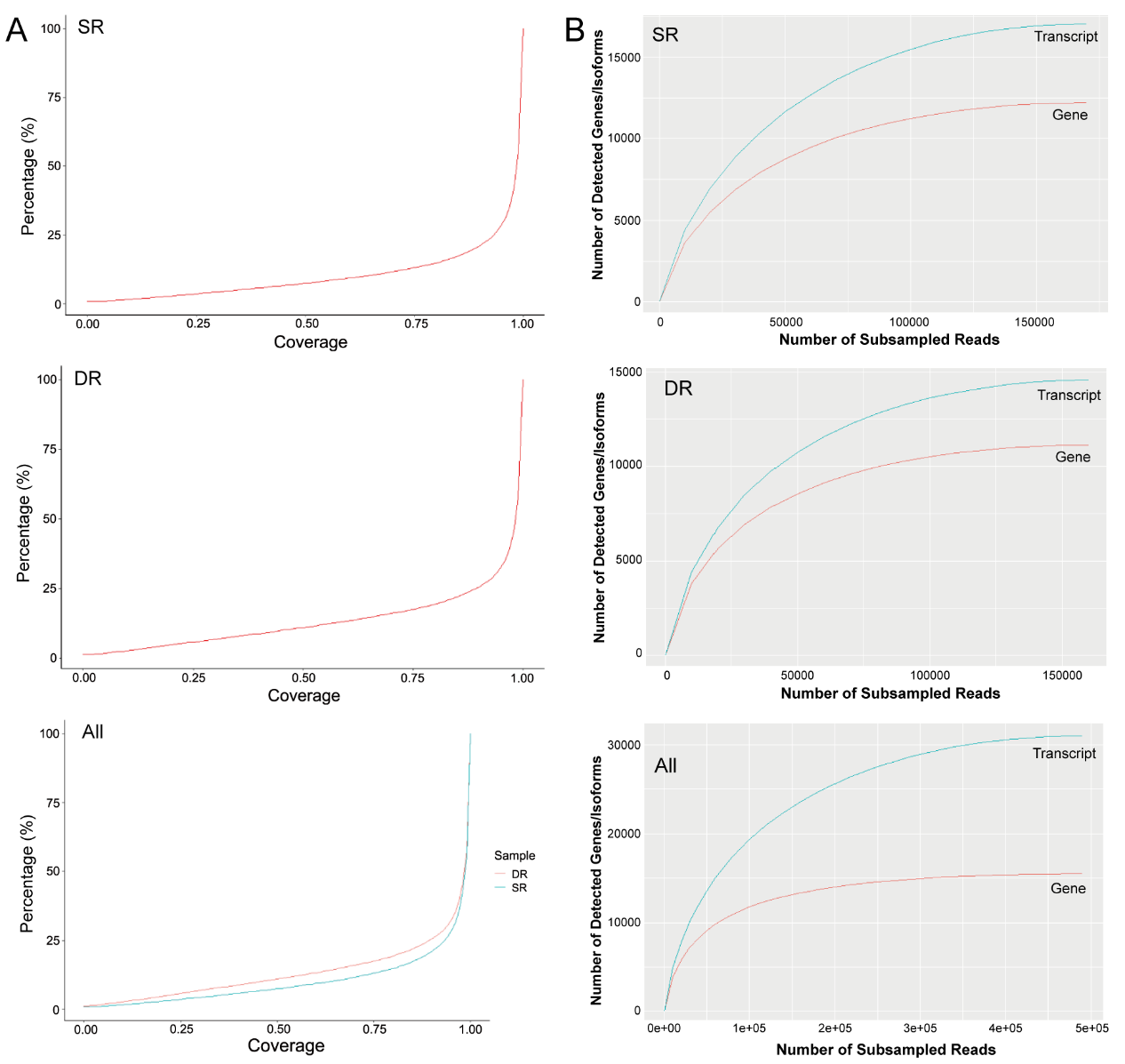
Supplementary figure S3**: Quality assessment of non-redundant FLNC transcript databases. (A). Coverage of RNA-seq in the All non-redundant FLNC transcripts database. To assess the quality of iso-seq data, RNA-Seq data were mapped to the All non-redundant FLNC transcripts database of the hybrid build, and their coverage was then calculated. 79.01% of SRs had RNA-Seq data coverage greater than 0.9, and 7.67% had coverage equal to 1; 74.52% of DRs had RNA-Seq data coverage greater than 0.9, and 7.61% had coverage equal to 1. The horizontal coordinates indicate the magnitude of coverage and the vertical coordinates indicate the proportion of RNA-Seq data volume, in All the red line indicates the DR group and the blue line indicates the SR group. (B). Unigenes - transcripts saturation curves for non-redundant FLNC transcripts. The saturation curve assesses the relationship between the amount of iso-seq sequencing data and the number of Unigenes measured in UniTransModels by calculating the number of different numbers of full-length transcripts measured in relation to the number of Unigenes measured in UniTransModels. The horizontal coordinate is the number of full-length reads and the vertical coordinate indicates the number of Unigenes or transcripts, with the red line indicating the number of Unigenes and the blue line indicating the number of transcripts. As the number of reads increases, the saturation curve for Unigenes and transcripts flattens out.


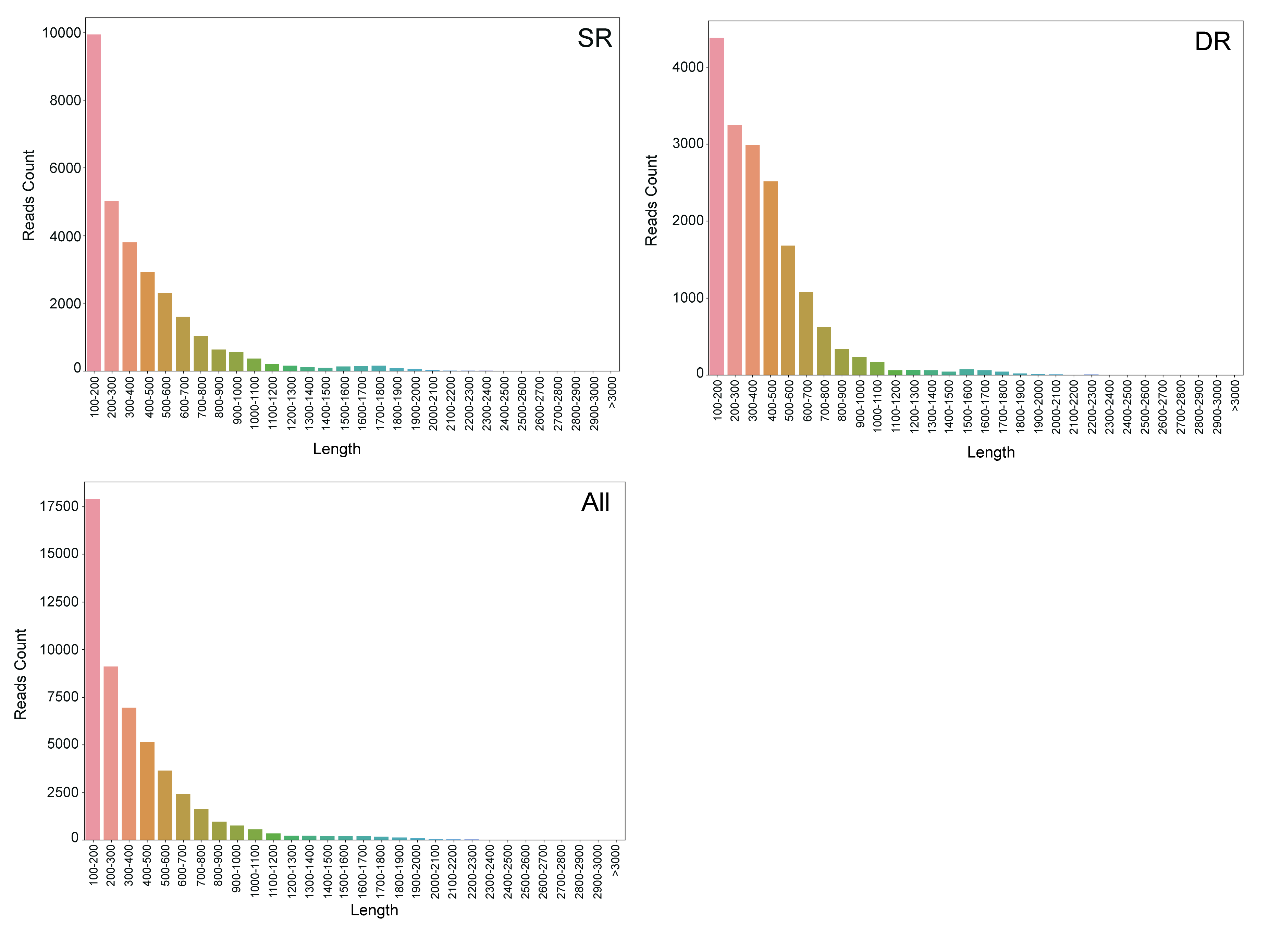


**Supplementary figure S4**: Length distribution of predicted candidate CDS sequences in the full-length transcriptome.

**
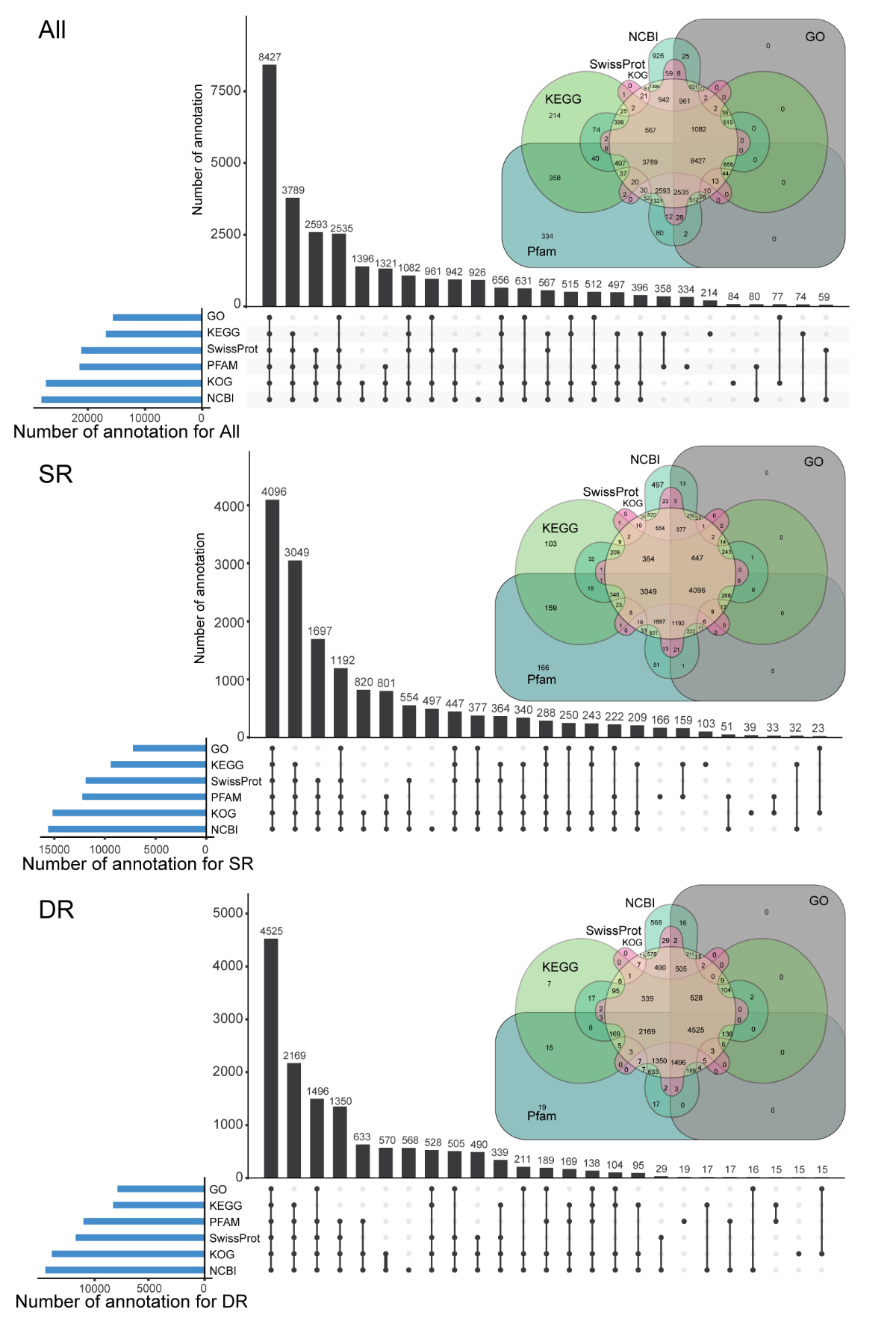
**

**Supplementary figure S5**: Upset plots of annotation results for three full-length non-redundant transcript databases.

**
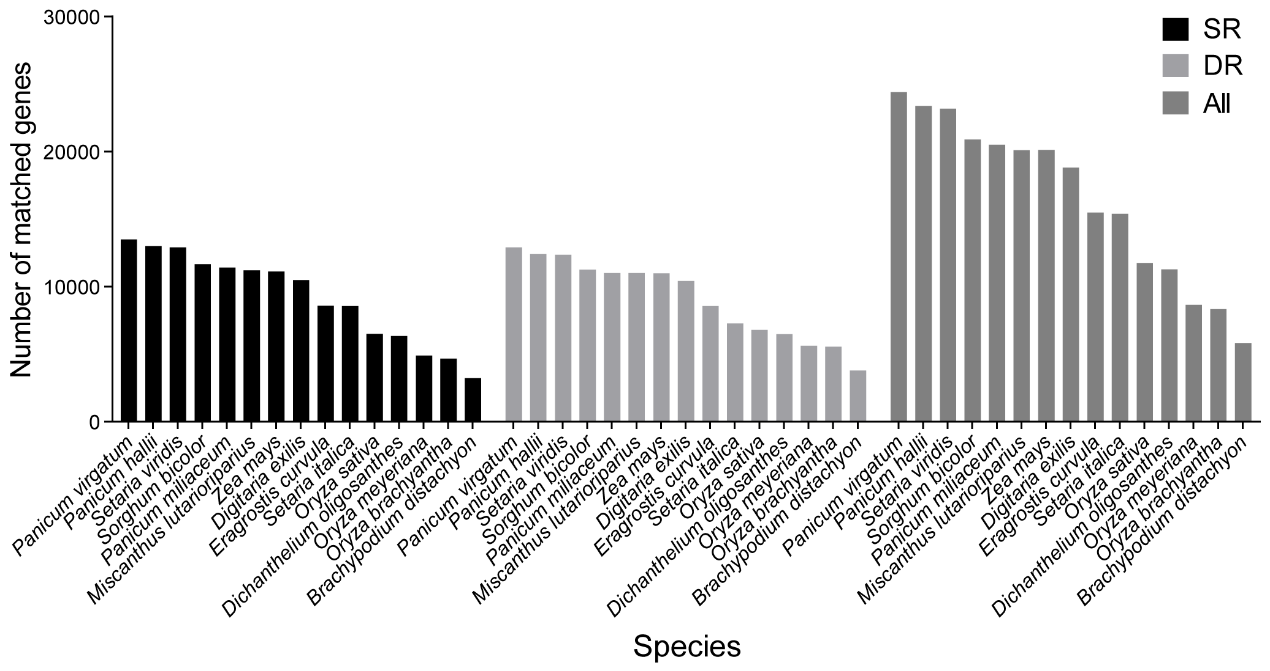
Supplementary figure S6**: Distribution of homologous species in the SR, DR and All non-redundant transcriptomes annotated in the NCBI non-redundant protein sequences database.


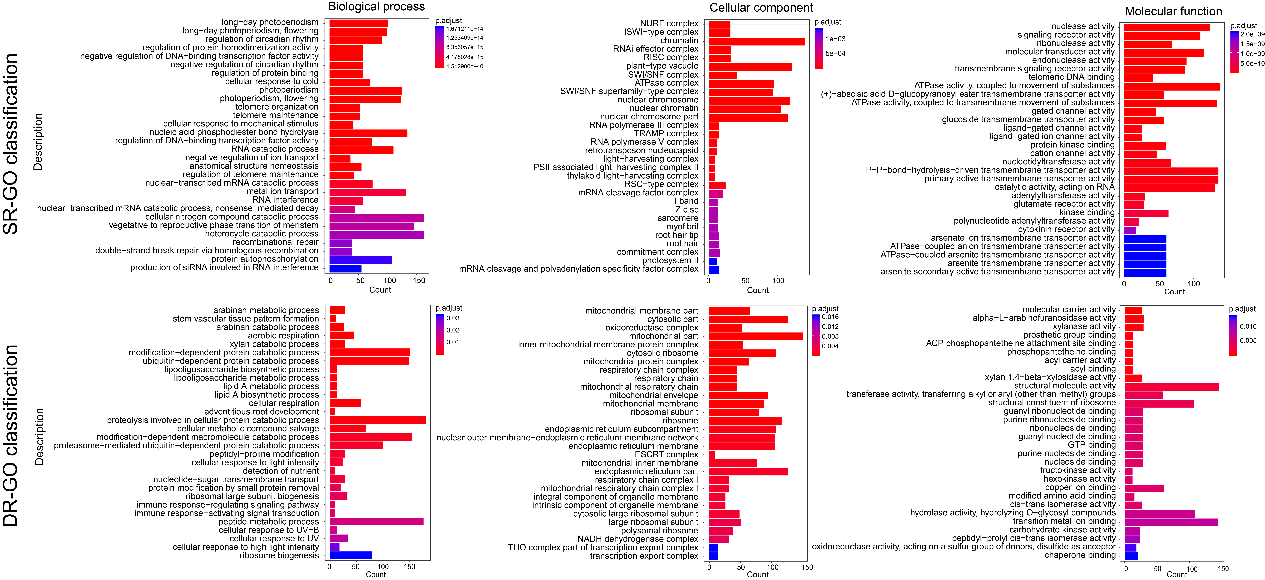


**Supplementary figure S7**: GO function clustering results for DR specific-unique transcripts. The x-axis is the number of transcripts. The y-axis is the function description, arranged according to the significance results.The color keys represent the statistical significance (*p.adjust*) size of the clustering results.

**
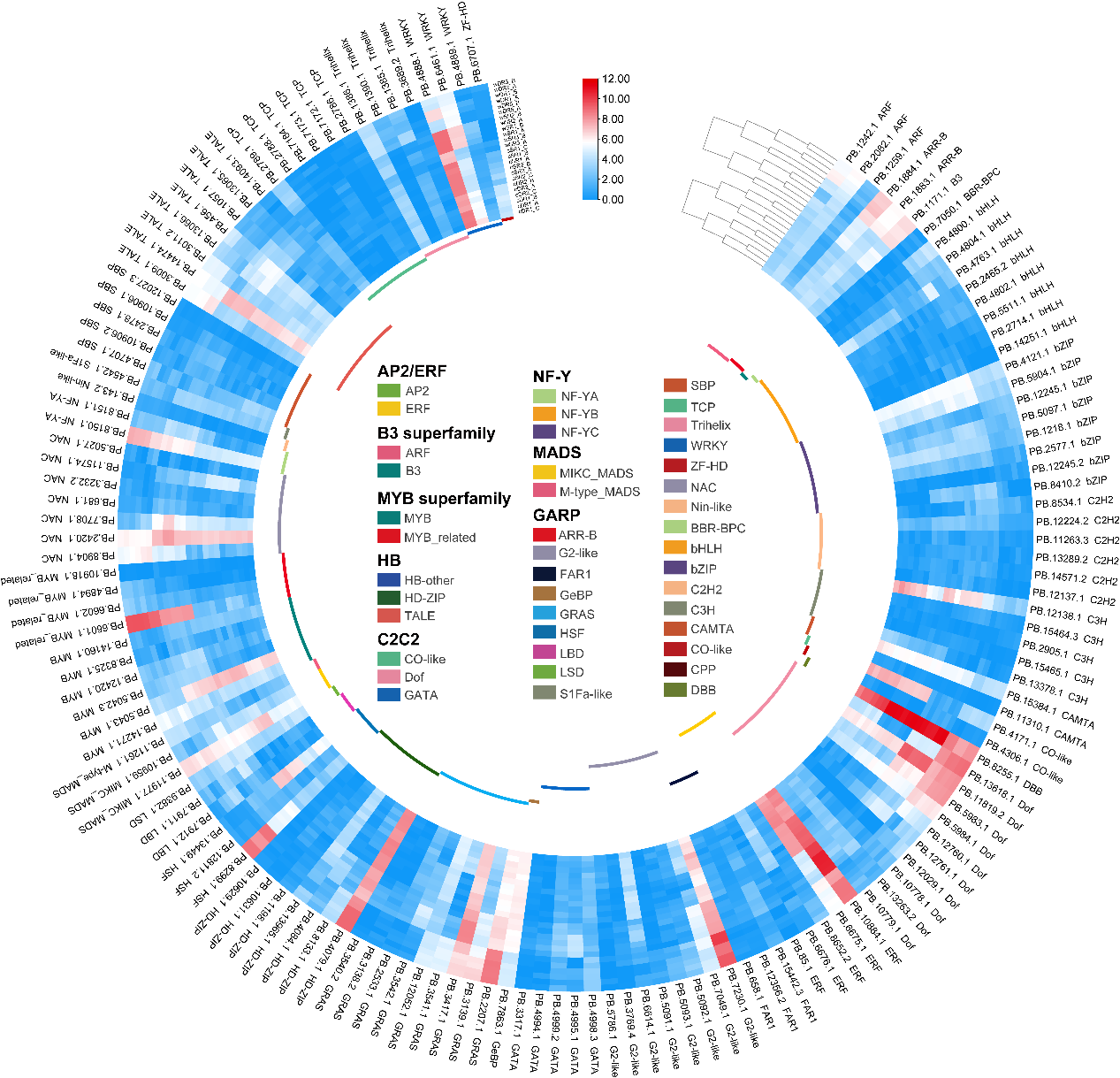
Supplementary figure S8**: Differentially expressed genes in transcription factors identified in *Phragmites australis*.


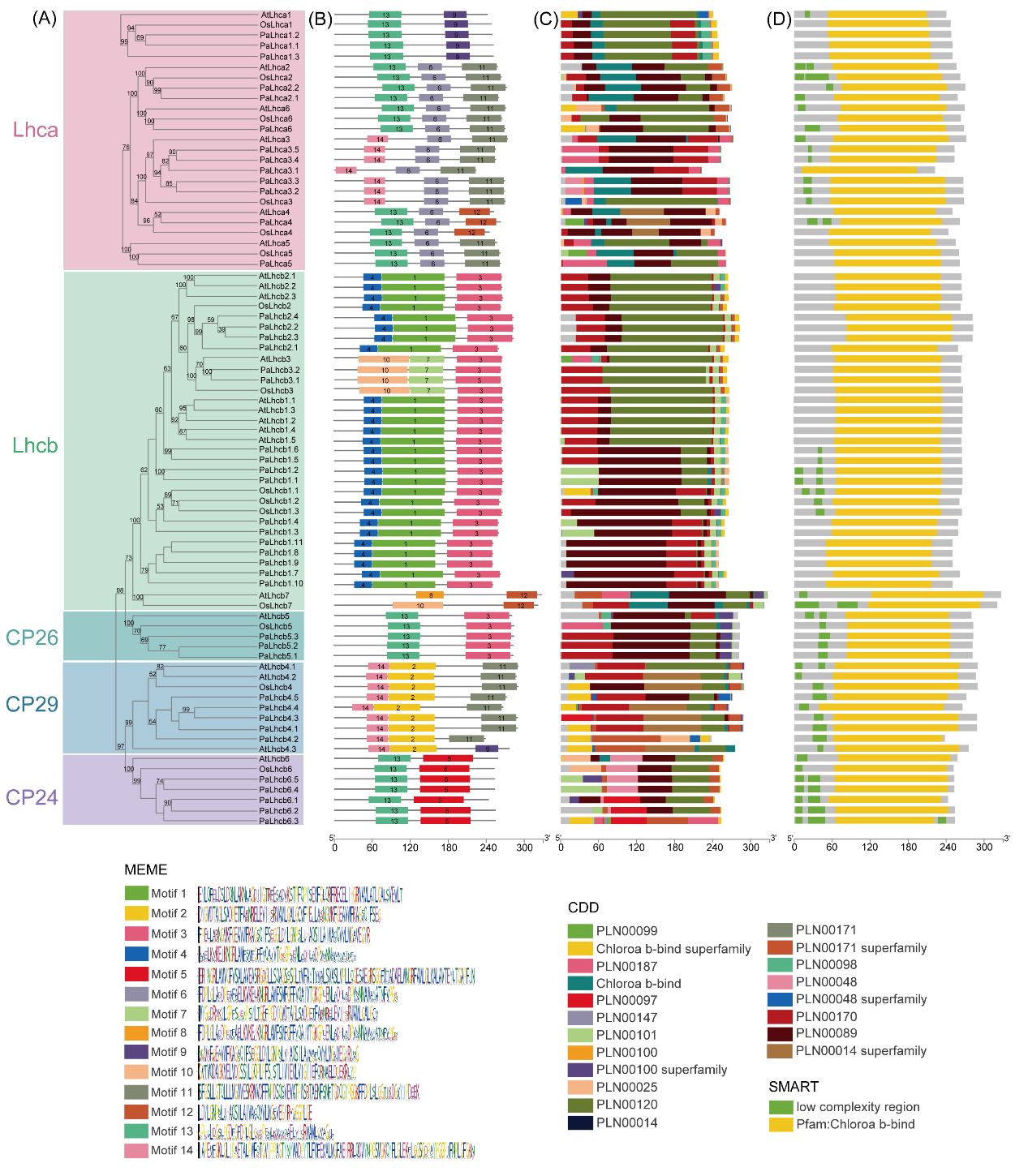


**Supplementary figure S9**:Structural analysis of Lhc family proteins in *Phragmites australis* (43), *Arabidopsis thaliana* (21), and *Oryza sativa* (15). (A). Phylogenetic analysis of Os/At/PaLhc family proteins; (B). Distribution of conserved motifs; (C). CDD predicted structural domain results; (D). SMART predicted structural domain results. Sequence comparisons were performed using mafft and phylogenetic trees were constructed using iq-tree (maximum likelihood method; bootstrap, 1000 replicates, only bootstrap values at nodes supported by a posterior probability of ≥50% are given). Conserved motifs were identified by MEME. c and d are structural domains in predicted protein sequences using CDD and SMART, respectively; See Materials and methods for detailed parameters. TBtools software was used to visualize the structures.
